# Locally Reprogramming Tumor-Associated Macrophages with Cytokine-Loaded Injectable Cryogels for Breast Cancer

**DOI:** 10.1007/s10439-025-03823-x

**Published:** 2025-08-29

**Authors:** Sydney R. Henriques, Evan B. Glass, Kristen L. Hoek, Ori Z. Chalom, Abigail E. Manning, Sohini Roy, Diana K. Graves, Sarah M. Goldstein, Benjamin C. Hacker, Renjie Jin, Marjan Rafat, Paula J. Hurley, Laura C. Kennedy, Young J. Kim, Andrew J. Wilson, Fiona E. Yull, Todd D. Giorgio

**Affiliations:** 1 Department of Biomedical Engineering, Vanderbilt University, Nashville, USA; 2 Department of Pharmacology, Vanderbilt University, Nashville, USA; 3 Department of Otolaryngology, Vanderbilt University Medical Cente, Nashville, USA; 4 Johnson and Johnson, Cambridge, USA; 5 Department of Pathology, Microbiology and Immunology, Vanderbilt University, Nashville, USA; 6 Department of Chemical and Biomolecular Engineering, Vanderbilt University, Nashville, USA; 7 Department of Medicine, Vanderbilt University Medical Center, Nashville, USA; 8 Vanderbilt Ingram Cancer Center, Nashville, USA; 9 Regeneron Pharmaceuticals, New York USA; 10 Department of OB/GYN, Vanderbilt University Medical Center, Nashville, USA; 11 Program in Cancer Biology, Vanderbilt University, Nashville, USA

**Keywords:** Cryogels, Macrophages, Immunotherapy, Cytokines, Breast cancer, Tumor microenvironment

## Abstract

**Purpose:**

Tumor-associated macrophages (TAMs) are the most abundant immune cells in primary solid tumors, including breast cancer, and typically exhibit an M2-like, immunosuppressive phenotype that promotes tumor growth. Given that TAMs can be repolarized through cytokine signaling, we propose a localized cytokine delivery depot using an injectable alginate cryogel to reprogram TAMs and create an inflammatory, anti-tumor TME.

**Methods:**

The cryogels were fabricated using cryogelation to generate a macroporous structure, followed by ionic crosslinking to enhance mechanical integrity while preserving pore size distribution. In vitro studies were conducted using bone marrow-derived macrophages, tumor-associated macrophages, and tumor explants. In vivo studies were conducted by orthotopically implanting breast tumors in the fat pads of FVB mice. Cell makeup and tissue composition were analyzed using qRT-PCR, flow cytometry, and Luminex panels. Statistical significance was determined using ANOVA and *t*-tests.

**Results:**

In vitro, cryogels released chemokines and cytokines, attracted M2 macrophages, and repolarized them toward M1-like activities. In vivo, cryogel treatment increased the presence of M1 macrophages relative to M2 macrophages in both the primary tumor and lungs, reduced primary tumor growth, and decreased T-cell exhaustion.

**Conclusions:**

A localized, injectable cryogel depot successfully induces an inflammatory TME, leading to reduced tumor burden and T-cell exhaustion while avoiding systemic toxicities associated with cytokine delivery.

**Supplementary Information:**

The online version contains supplementary material available at 10.1007/s10439-025-03823-x.

## Introduction

More than 250,000 women develop breast cancer in the United States annually, with over 40,000 fatalities [1]. Breast cancer is currently the most prevalent form of cancer in the United States, and since 2012, the rates of breast cancer in women have increased by 1% per year [2]. The standard of care for solid tumors includes surgery, chemotherapy, and radiation. However, these treatments generally impact non-tumor tissues and can result in severe adverse side effects.

Cancer immunotherapy is a strategy that leverages the immune system to generate an anti-tumor response, and it primarily consists of immune checkpoint blockade (ICB) that enables anticancer T-cell responses and chimeric antigen receptor (CAR) T-cell therapies [3–5]. Successful immunotherapies control tumors and their metastatic spread, while minimizing side effects common in other anticancer therapies like chemotherapy and radiation. Unfortunately, a significant fraction of patients are unresponsive or poorly responsive to these treatments, especially in the context of solid tumors [3, 6–8].

Inducing an anti-tumor immune response in solid tumors depends on overcoming the immunosuppressive tumor microenvironment (TME) that limits T-cell infiltration and produces signals that cause nearby T cells to become dysfunctional and inactive, thereby inducing T-cell exhaustion [9, 10]. Inadequate populations of tumor-resident T cells combined with a lack of proven strategies to increase T-cell populations in the TME represent a significant obstacle to successful cancer immunotherapy. Evidence suggests that macrophages, the most abundant immune cell within most solid tumors, can be leveraged to promote an anti-tumor response and enable T-cell-mediated cancer cell killing [11–13].

Macrophages exist on a spectrum of phenotypes, but are often simplified into two extremes: pro-inflammatory M1-like and immunosuppressive/wound-healing M2-like macrophages [14, 15]. A higher ratio of M1:M2-like tumor-associated macrophages (TAMs) in breast cancer patients is associated with a favorable outcome [16]. Therefore, quantifying the M1:M2 ratio of TAMs can be a useful prognostication tool [17]. Yet, in the TME, TAMs are often strongly skewed toward M2-like functions that promote tumor growth and release immunosuppressive signals that restrict other immune cells from recognizing and killing the cancer cells [18–21]. Reprogramming TAMs to be pro-inflammatory is hypothesized to result in a TME that is “primed” for anticancer immunity [22, 23]. Repolarizing TAMs is possible due to the phenotypic plasticity that can be achieved via the delivery of inflammatory cues, such as cytokines [24]. Therefore, we and others hypothesize that increasing M1-like functions in TAMs could generate an inflammatory TME that supports anti-tumor responses, recruits T cells to tumors, and provides an opportunity to enable effective immunotherapy [22, 23].

IL-12 and IFN-γ are well-established cytokines that play critical roles in macrophage polarization toward an M1-like state [25, 26]. IFN-γ directly activates macrophages, promoting the expression of genes associated with the M1 phenotype, including those involved in producing inflammatory cytokines and reactive oxygen species [27]. IL-12 stimulates the production of IFN-γ from macrophages, as well as natural killer (NK) cells and T cells, creating a positive feedback loop that promotes M1 polarization. IL-12 itself can also polarize macrophages toward an M1-like state [26]. However, repolarizing TAMs with systemic cytokine delivery is limited by widespread inflammation and off-target toxicity. Side effects of systemically delivered IL-12 and IFN-γ include splenomegaly, leukopenia, gastrointestinal symptoms, and flu-like symptoms [28, 29]. To minimize off-target effects, we propose the localized delivery of cytokines to the tumor via an injectable biomaterial scaffold. Injectable biomaterials solve unique challenges for a variety of disease applications, as detailed in recent reviews [30, 31]. One intriguing example is the development of a tough, macroporous alginate hydrogel that withstands injection via syringe. Hydrogels have previously been used as subcutaneous delivery depots of biologics and allow for their controlled release [32]. These hydrogels, or "cryogels," are created at − 20 °C to form macroscopic pores and then can be loaded with cell-signaling molecules for localized delivery into the TME [33, 34]. In addition to the initial covalent crosslinking, these cryogels can be ionically crosslinked to form a tough cryogel capable of being injected through a syringe while maintaining overall pore size and structure [34]. The high-water content of hydrogels makes them optimal materials to protect biologics against degradation [35]. Cryogels injected subcutaneously have been used as artificial lymph nodes [34, 36]. This strategy, however, fails to modulate the immunosuppressive TME that limits the migration of T cells from the subcutaneous cryogel and consequent tumor infiltration. Here, we peritumorally inject a cryogel loaded with macrophage-specific chemokines and inflammatory cytokines to decrease immunosuppression in the proximal TME.

To home M2 macrophages, which are the predominant myeloid population in the tumor periphery [37], toward the cryogel, we deliver chemokine (C-C motif) ligand 2 (CCL2). CCL2 binds to the CCR2 receptor on macrophages, promoting their migration up the CCL2 concentration gradient [38]. In the current approach, CCL2 is intended to attract M2-like macrophages located in the tumor periphery toward the cryogel, where they will come into contact with interleukin-12 (IL-12) and interferon gamma (IFN-γ) and be polarized toward M1-like characteristics. Thus, IL-12 and IFN-γ release from the cryogel that is adjacent to the tumor enables TAM reprogramming and reversal of the immunosuppressive TME while avoiding dose-limiting toxicities of systemically administered pro-inflammatory cytokines.

The cryogel is sufficiently large to avoid the biodistribution concerns associated with injection of nano- or microparticles. Localized injection next to the tumor also implies that the delivered proteins are less prone to the rapid clearance that is often observed with systemically administered nano- or microparticle drug delivery systems. The macroporous structure of the cryogel offers an advantage of allowing immune cells to infiltrate, potentially facilitating extended immune engagement at the deposition site next to the tumor. This cryogel system is intended to provide minimally invasive treatment of solid tumors while also reducing the risk of toxicity that is associated with systemic administration of pro-inflammatory cytokines.

We aim to develop an injectable, localized cytokine delivery depot to shift TAM behavior toward an anti-tumor phenotype that results in decreased immunosuppression and increased T-cell responses. This approach is hypothesized to induce an inflammatory TME capable of inhibiting tumor progression.

## Materials and Methods

### Animals

All animal studies were approved by the Vanderbilt University Institutional Animal Care and Use Committee (IACUC). FVB/NJ male and female mice were obtained from The Jackson Laboratory. To generate female mice with spontaneous mammary tumors, female FVB mice were crossed with male mouse mammary tumor virus-polyoma middle T antigen (MMTV-PyMT) transgenic mice (from Dr. Fiona Yull, Vanderbilt University, Nashville, TN). For in vivo studies, female FVB mice between the ages of 8 and 12 weeks were used for orthotopic injections of the PyMT-17L3C cell line (from Dr. Barbara Fingleton, Vanderbilt University, Nashville, TN) [39].

### Cell Culture

Bone marrow-derived macrophages (BMDMs) were isolated from the femurs and tibias of female FVB mice and cultured as previously described [40]. BMDMs were cultured in L929-conditioned media: Dulbecco’s Modified Eagle Medium (DMEM, Gibco; 15-018-CV) supplemented with 10% fetal bovine serum (FBS, Gibco; 26140079), 1% penicillin–streptomycin (P/S, Corning; MT30002CI), 1% L-glutamine (Sigma-Aldrich; G7513), and 14% 1:1 (v/v) L929 week 1 and week 2 media. L929 media was prepared as previously described [40]. BMDMs were treated with 10 ng/mL IL-4 (R&D Systems; 404-ML-010/CF) and 20 ng/mL IL-13 (R&D Systems; 413-ML-005/CF) for four days for M2 polarization or 100 ng/mL IFN-γ (BioLegend; 575304) and 0.1 ng/mL lipopolysaccharide (LPS, Sigma-Aldrich; L2630) for 24 h for M1 polarization. Unpolarized (M0) BMDMs received plain BMDM media. All subsequent treatments were performed after macrophage polarization.

PyMT-17L3C cells were cultured in DMEM supplemented with 10% FBS, 1% P/S, and 1% L-glutamine as well as 10 µL 1 mg/mL Puromycin (Sigma; P8833) to maintain the luciferase construct. All cell lines tested negative for mycoplasma contamination (MycoAlert Mycoplasma Detection Kit, Lonza; LT07-318).

### Fabrication of MA-Alginate

MA-Alginate was fabricated as previously described [34]. Briefly, 1.0 g UP MVG sodium alginate (NovaMatrix, Sandvika, Norway) was slowly added to a 0.1 M 2-(N-morpholino) ethanesulfonic acid (MES) buffer (pH = 6.4) at 0.6% (w/v). The alginate was dissolved in a glass vial on a stir plate to generate a homogeneous solution. To activate the carboxyl groups on the alginate backbone, NHS (1.3 g) and EDC (2.8 g) were added and allowed to stir for 5 min. AEMA (2.24 g, Sigma-Aldrich) was added and the reaction vessel was covered and left to stir for 24 h at room temperature. The alginate solution was then precipitated dropwise into excess acetone and filtered through a 0.45 µm filter to collect the solid MA-alginate. The resulting MA-alginate was dried in a vacuum oven overnight at RT. To purify the final product, the MA-alginate was dissolved at 1% (w/v) in deionized (DI) water and dialyzed against DI water for 3 days (MWCO = 3500 kDa). The final solution was collected, frozen at −80 °C, and lyophilized. For NMR analysis, MA-alginate was dissolved at 1% (w/v) in deuterium oxide (D_2_O).

### Cryogelation of MA-Alginate

Cryogels were fabricated as previously described [33, 34]. To form cryogels, 10 mg MA-alginate was dissolved slowly at 1.5% (w/v) in DI water (667 µL) on a stir plate. The dissolved MA-alginate was then cooled for > 1 h at 4 °C. Radical initiators N,N,N′,N′-tetramethylethylenediamine (TEMED) (0.5% w/v) and ammonium persulfate (APS) (0.25% w/v) were added and the solution was immediately pipetted into a precooled (− 20 °C) PTFE mold and placed at − 20 °C. For all gel injection and cell culture experiments, cryogels were designed to be circular with a 5 mm diameter and 1 mm thickness. For physical characteristic tests, including rheology measurements, square cryogels with 5 × 5 × 2 mm dimensions were used. For loaded cryogels, IFN-γ, IL-12, and CCL2 were added to the gelation solution at a final mass of 100 ng or 500 ng of each protein per gel. The gels were crosslinked for 24 h before thawing. The cryogels were rinsed with DI water to remove excess initiators and then ionically crosslinked in a 200 mM calcium chloride solution for 10 min to form “tough” cryogels. For NMR samples, the MA-alginate was dissolved in D_2_O, mixed with radical initiators, and directly added to an NMR tube and left at − 20 °C overnight.

### Physical Characterization of Cryogels

For SEM, cryogels (control or injected) were dehydrated in serial dilutions of ethanol in DI water. Gels were soaked in 30, 50, 70, 90, and 100% ethanol in DI water (v/v) for 20 min each. The fully dehydrated cryogels were then soaked in hexamethyldisilazane (HMDS) for 10 min and placed under vacuum for one h. For cryosectioning, fully dried cryogels were placed in optimal cutting temperature (OCT) compound and frozen at −80 °C. The frozen gels were then cryosectioned into 20 µm slices. Samples (whole gels or sections) were then placed on SEM specimen mounts with double-sided carbon tape. Sectioned gels were rinsed briefly with DI water to remove OCT and dried overnight in a vacuum oven. All samples were sputter-coated with Au and imaged on a Zeiss Merlin SEM in the Vanderbilt Institute for Nanoscale Science and Engineering (VINSE). Magnification ranged from 115 ×  to 2.45 k  × , depending on the image. A voltage of 2 kV was used, and the working distance was set between 4.9 and 6.1 mm. Specifics for each image can be found on individual SEM images.

To quantify hydrated pore size, cryogels were labeled with Cy5-amine to create fluorescent gels. Briefly, cryogels were submerged in a 0.1 M 2-(N-morpholino)ethanesulfonic acid (MES) buffer (pH = 6.5) at 1 mL/gel. NHS (7.8 mg) and EDC (16.8 mg) were added and mixed on a shaker for 10 min to activate the carboxyl groups on the alginate backbone. After activation, Cy5-amine (6.9 µg, Cat. #130C0, Lumiprobe, Maryland, USA) was added, and the solution was placed on a shaker for 2 h in the dark. Excess Cy5-amine was washed off with DI water, and gels were placed on a microscope slide and covered with a few drops of Hank’s Balanced Salt Solution (HBSS) to prevent dehydration. Fluorescent imaging was conducted on a Nikon Czsi + system with a Nikon Eclipse TioE inverted microscopy base, Plan ApoVC 20× differential interference contrast N2 objective, and a 505/565 dichroic mirror. All image acquisition was performed using Nikon NIS-Elements AR version 4.50.00. Image processing and analysis were done using Fiji in ImageJ version 1.53f51 [41].

All other physical characterizations were performed with square cryogels. To determine the interconnected porosity and swelling ratio, we employed the wicking method [33]. Gels were hydrated in DI water for 2 min before measuring the hydrated mass (*m*_*h*_). Excess water was wicked away with a kimwipe before weighing dehydrated gels (*m*_*d*_). A kimwipe was used as it quickly dehydrated the cryogels without adhering to them. Interconnected porosity (*P*) was calculated as (*m*_*h*_ − *m*_*d*_)/*m*_*h*_ × 100%. For swelling ratio, gels were serially dried in ethanol as done previously before placing under vacuum for 24 h. The fully dried gels were weighed (*m*_*dry*_) and swelling ratio calculated as *m*_*h*_/*m*_*dry*_. Rheology measurements were performed with a rheometer to determine Young’s modulus (E) of the cryogels. An amplitude sweep was performed to determine storage modulus (G′) and loss modulus (G″). The temperature was kept at 25 °C, the frequency was 1 Hz, and the strain ranged from 4.7% to 4.95%. These values were then used to calculate the shear modulus (G) using G = $\sqrt{({G{\prime}}^{2}+{G^{\prime\prime} }^{2})}$. Young’s modulus was then calculated as E = 2 × G(1 + ν) where Poisson’s ratio (ν) was taken to be 0.5, which is common for hydrogel characterization [42].

### Viability Assays for BMDMs

Free cytokine treatments (unformulated, not encapsulated in a cryogel) were conducted at concentrations of 100 or 500 ng/mL of IFN-γ (BioLegend; 575304) or IL-12 (R&D Systems; 419-ML-010/CF) for 48 h, and the co-treatment was performed at 100 ng/mL each of IFN-γ and IL-12 for 48 h. To assess viability, a CellTiter-Glo® Luminescent Cell Viability Assay (Promega; G7571) was used. For cryogel treatments, the fabricated cryogels were moved to a 12-well plate and placed under ultraviolet (UV) light for 30 min to sterilize. One sterilized gel was added per well in 12-well plates with polarized BMDMs for 48 h. At endpoint, the cell supernatant was used for CellTiter-Glo® viability assay.

### CCL2-Induced Migration of BMDMs

CCL2-induced M2 BMDM migration was evaluated using a μ-Slide Chemotaxis plate (Ibidi®, Germany). After polarization, 6 μL of 2.5 × 10^6^ cells/mL M2 BMDMs was plated in the cell channel and incubated overnight to allow cells to adhere. The cell channel was washed twice with media containing 2% FBS and one chamber for each sample was filled with 60 µL 2% FBS media. The other chamber was filled with either 2% FBS (control) or varying concentrations of CCL2 in 2% FBS media. The chemotaxis plates were immediately placed in an incubation chamber under a Leica DMi8 microscope and imaged every 30 min for 24 h. Cell migration was tracked via ImageJ Manual Tracking plugin by counting at least 20–30 cells/image with three sections per treatment condition. These data were then analyzed using the Chemotaxis and Migration Tool provided by Ibidi. To quantify chemokine-induced cell migration, the change in center of mass in the direction of the chemokine gradient (COMD) was reported at timepoints of 6, 12, and 24 h [43].

For all cryogel migration studies, empty and loaded cryogels were labeled with Cy5-amine and sterilized as previously described. To evaluate chemoattraction, Cy5-gels were placed on sterilized coverslips in six-well plates. Macrophages were added dropwise onto the gels (500,000 cells/well in 2 mL media) and the cells were incubated for 48 h. The cells were fixed with 5% formalin for 20 min at RT before washing 3× in a wash buffer (0.1% BSA w/v in PBS). The slides were then covered with a blocking buffer consisting of 5% goat serum (Abcam; ab7481) v/v in PBS and incubated at RT for 45 min to prevent nonspecific antibody labeling. The blocking buffer was removed, and 100 µL of 1:100 dilution of rat anti-mouse F4/80 (Bio-Rad; MCA497GA, Clone Cl:A3-1) was added and incubated at 4 °C overnight. The cells were washed again before permeabilizing with 0.5% Triton X100 in PBS (−/−) for 5 min at RT. The cells were stained with Hoechst at 1 μg/mL for 5 min at RT in the dark. ProLong Gold anti-fade mounting media (ThermoFisher; P10144) was added to the coverslip before placing it on a microscope slide and imaging with a Nikon Czsi + system with a Nikon Eclipse TioE inverted microscopy base, Plan ApoVC 20× differential interference contrast N2 objective, and a combination of 405/488 and 505/565 dichroic mirrors. Image analysis was performed using ImageJ.

### Establishing Cryogel Release Kinetics

Cryogels were prepared as previously described and loaded with 100 ng/cryogel of IFN-γ, IL-12, and CCL2. Cryogels were stored at 37 °C in 1 mL PBS with 0.1% BSA. At specified timepoints, cryogels were centrifuged, and the supernatant was removed and stored at −20 °C until further use. An enzyme-linked immunosorbent assay (ELISA) kit (R&D, DY008B, DY485, DY419, DY479) was used to quantify the amount of cytokine and chemokine in each sample.

### Treating Isolated Tumor-Associated Macrophages or Whole Tumor Explants

Female PyMT mice spontaneously develop tumors in mammary fat pads, which can be collected once palpable for single-cell isolation. Tumors were dissected before becoming necrotic (size < 15 mm) and cut into small pieces. The tumor tissue was dissociated using a gentleMACS^TM^ Dissociator (130-093-235; Miltenyi Biotec) and the corresponding Tumor Dissociation Kit (Cat. #130-096-730, Miltenyi Biotec). After tissue dissociation, the samples were filtered through a 70 µm MACS^®^ SmartStrainers (130-098-462; Miltenyi Biotec) and centrifuged at 300 g for 7 min. Blood cells were lysed as described previously, and the resulting solution was recentrifuged. TAMs were isolated via CD11b magnetic bead separation by binding cells with CD11b magnetic beads (130-049-601; Miltenyi Biotec) and filtering them through LS columns (130-042-401; Miltenyi Biotec). Isolated TAMs were counted and plated at 500,000 cells/well in a 12-well plate and used for 48-h cryogel treatments as previously described.

To establish a comprehensive ex vivo model of the tumor microenvironment, PyMT tumor explants were cultured. Primary PyMT tumors were surgically harvested from PyMT mice and cut into small, 2–3 mm pieces. These explants were then placed in a 12-well plate with 1 mL media consisting of DMEM (15-018-CV) supplemented with 1% L-glutamine, 1% P/S, 1% MEM vitamins, and 10% FBS. The explants were treated with cryogels as previously described for 48 h.

### In Vivo Cryogel Treatment in Tumor-Bearing Mice

Luciferase-expressing 17L3C polyoma (PyMT-17L3C) cells were cultured, collected, and resuspended in 1 ×  PBS (−/−) at a concentration of 2 × 10^6^ cells/100 µL PBS. For tumor induction, 50 µL of cell suspension (1 × 10^6^ cells) was orthotopically injected directly into the #4 mammary fat pad of female FVB mice between the ages of 8 and 12 weeks. The tumors developed until palpable (~100 mm^3^) before treatment. Mice were randomly selected by cage and treated by peritumorally injecting 100 µL PBS (−/−) containing either a single Empty Gel or a single loaded gel through a 16G needle. For multi-gel treatments, this was repeated three times around the periphery of the tumor. At endpoint, all mice were euthanized following approved IACUC protocols using carbon dioxide inhalation followed by secondary cervical dislocation. Timelines of the in vivo experiments conducted in this paper can be seen in Supplemental Fig. S2. If mice had subcutaneous tumors (rather than orthotopic tumors), they were excluded from the analyses. Likewise, there were some instances where the tumor engulfed the lymph node; in these cases, these data points were not included in T-cell analyses.

### Quantitative Reverse Transcription Polymerase Chain Reaction (qRT-PCR)

RNA isolation, cDNA fabrication, and qRT-PCR analysis were performed as previously described [40, 44, 45]. For BMDMs and TAMs, RNA was isolated using the RNeasy Mini Kit (Qiagen; 74106). Residual DNA was removed using RNase-free DNase (Qiagen; 79256). cDNAs were synthesized using a SuperScript IV reverse transcriptase kit (Invitrogen; 18090050) and quantitative reverse transcription polymerase chain reaction (qRT-PCR) was performed using SsoAdvanced Universal SYBR Green Supermix (Bio-Rad; 1725270) on a CFX96 real-time PCR instrument and software (Bio-Rad) through the VUMC Molecular Cell Biology Resource (MCBR) core.

For tumor explants and in vivo tumor samples, tumor tissue was placed in TRIzol^TM^ (Invitrogen; 15596026) to lyse cells. Explant tissue was transferred to 2 mL tubes. Media in the wells was used to wash leftover cells off the plate into tubes, centrifuged at 1500 rpm for 5 min, and the supernatant was aspirated. Explants were then added to tubes containing 1 mL of TRIzol, vortexed, and stored at −80 °C. Samples were thawed, vortexed, and refrozen each day for 2–3 days before collecting RNA using Direct-zol RNA kits per the manufacturer’s instructions (R2050; Zymo Research).

### Flow Cytometry Analysis

For cultured BMDMs and TAMs, 1X Brefeldin (BioLegend; 420601) and 1X Monensin (BioLegend; 420701) were added to the cells for 12 h to preserve intracellular cytokines for flow cytometry. Cells were then washed with PBS (−/−) and trypsinized using 0.25% trypsin. Cells were collected, centrifuged at 300 × g for 5 min, and resuspended in 2 mL BMDM media for counting. After a second centrifugation, cells were resuspended in 500 µL PBS (−/−) and transferred to a 96-well plate. Cells were stained with live/dead exclusion dye and surface markers, then fixed and permeabilized for intracellular staining. The following anti-mouse antibodies were used in vitro, separated by supplier: BioLegend: CD11b (Clone M1/70), MHCII (Clone M5/114.15.2), CD206 (Clone C068C2), IL-10 (Clone JES5-16E3), IL-4 (Clone 11B11), TGF-Beta (Clone TW-16B4); BD Biosciences: TNF-alpha (Clone MP6-XT22), CD80 (Clone 16-10A1); Invitrogen: iNOS (Clone CXNFT), and IL-12 (Clone C15.6).

For in vivo studies, tumors were isolated, digested with 3 mg/mL collagenase, 0.03 mg/mL hyaluronidase, and 30 µg/mL DNase for 1 h in a shaking incubator at 37°, then passed through a 70 µm strainer in order to obtain single-cell suspensions. Cells were resuspended in complete media and counted. 1 × 10^6^ cells were transferred to a 96-well plate for staining. The cells were then stained for flow cytometry analysis as described previously. The anti-mouse antibodies used in vivo (separated by supplier) were as follows: Biolegend: CD11b (Clone M1/70), APC (Clone MB8), CD206 (Clone C068C2), MHCII (Clone M5/114.15.2), CD38 (Clone 90), F4/80 (Clone BMB), CD11c (Clone N418), Ly6C (Clone HK1.4), CD3 (Clone 145-2C11), PD1 (Clone RMP1-30), TIM3 (Clone B8.2C12), NK1.1 (Clone PK136), CD8alpha (Clone 53-6.7), LAG3 (Clone C9B7W); BD Biosciences: CD45 (Clone 30-F11), CD11c (Clone HL3), CD4 (Clone GK1.5) ; Invitrogen: CD163 (Clone TNKUPJ); and Cytek (formerly Tombo): Ly6G (Clone IA8), ghost dye violet 510, siglecF (Clone E50-2440), and B220 (Clone RA3-682). In vivo flow cytometry experiments were performed on either a BD LSRFortessa or a BD FACSCelesta. Flow analysis was performed using FlowJo software (Treestar). Flow gating strategy is shown in Supplemental Fig. S3 for in vitro studies and in Supplemental Figs. S4 and S5 for in vivo studies.

### Statistical Analysis

All statistical analyses were performed using a one-way ANOVA with Sidak’s multiple comparison test in the case of two or more groups, or a two-tailed Student’s *t*-test in the case of only two groups. All tests were performed with α = 0.05. Statistical analyses were performed using GraphPad Prism v10.2.0.

## Results

### Fabricating and Characterizing Injectable Cryogels

**Fig. 1:**
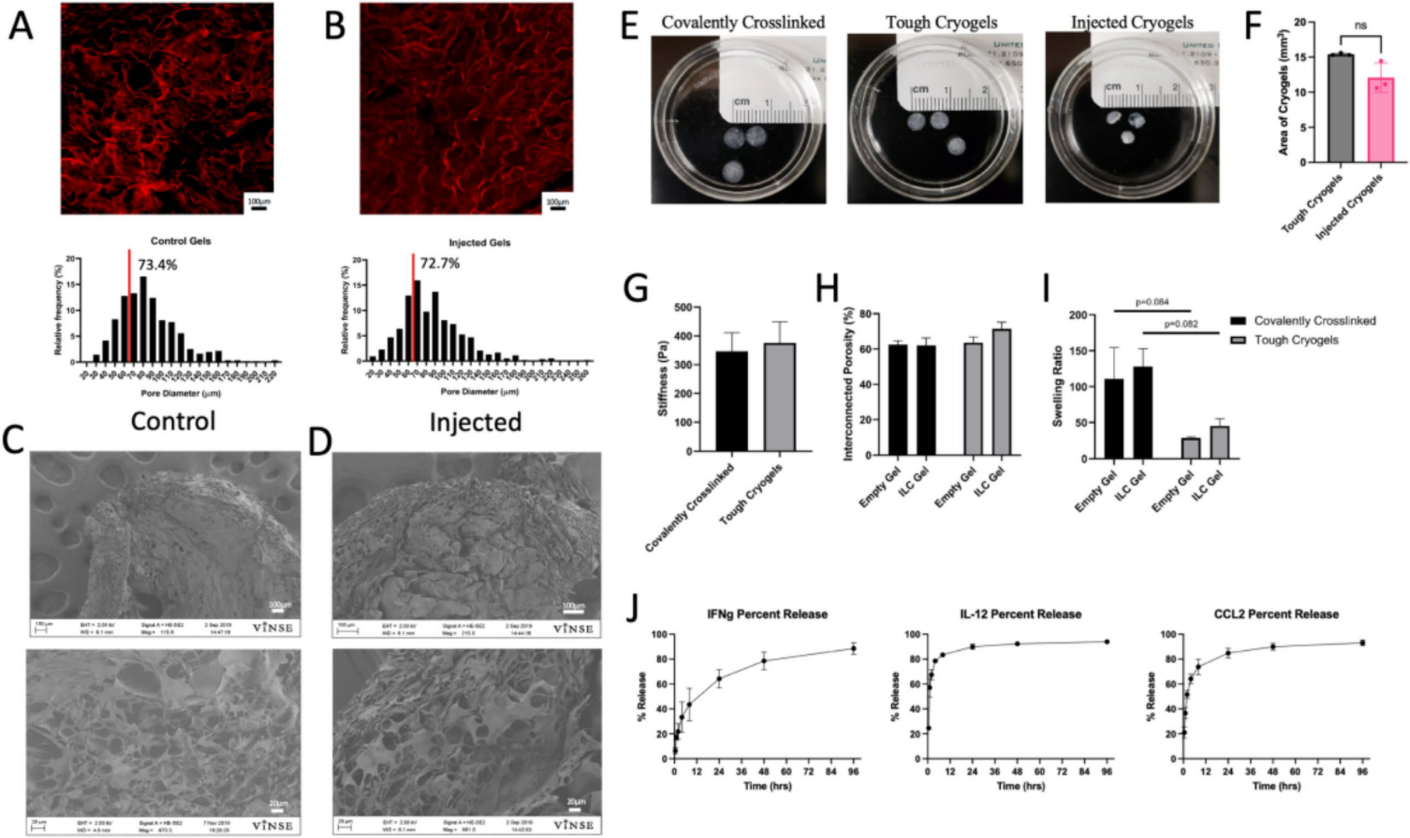
Tough cryogels maintain structure post-injection and display first-order release kinetics. **A** and **B** Control and Injected cryogels were labeled with a Cy5-amine and imaged with fluorescent microscopy. Pore measurements were made on hydrated samples in ImageJ and quantified for pore size distribution before and after injection. **C** and **D** Scanning electron microscopy confirmed no structural changes occurred post-injection. **E** Images of the covalently crosslinked cryogels, tough cryogels, and injected cryogels showed structural integrity was maintained post-injection, and (**F**) their area was significantly changed post-injection. **G**–**I** Mechanical characterization of cryogels shows gel stiffness did not change before and after ionic crosslinking (G). It also reveals that interconnected porosity (H) and swelling ratio (I) do not change between empty and loaded gels, but the swelling ratio is affected by ionic crosslinking forming tough cryogels. **J** Evaluation of the cumulative release of proteins at various timepoints revealed similar burst release profiles for IFN-γ, IL-12, and CCL2. All three molecules released greater than 89% of loaded cargo by 96 h of incubation

Sodium alginate was functionalized with aminoethyl methacrylate (AEMA) to form methacrylated alginate (MA-alginate), with crosslinking confirmed by 1H NMR spectroscopy (Fig. S6). Cryogelation in Teflon molds produced circular cryogels (5 mm diameter, 1 mm thickness) (Fig. 1E). Covalent crosslinking of methacrylates was performed at −20 °C to produce macroporous cryogels. Ionic crosslinking post-thaw in CaCl_2_ creates shear-thinning, or ‘tough,’ cryogels that can be injected through a 16G syringe [34].

Cryogels labeled with Cy5-amine were imaged by fluorescent confocal microscopy to estimate hydrated pore diameter. ImageJ quantification confirmed that 73.4% of control gel pores were over 70 µm, enabling the potential for cell infiltration (Fig. 1A and B). The SEM and confocal microscopy illustrate the ability of ionically crosslinked cryogels to withstand injection through a 16G needle while maintaining overall structure and pore size (Fig. 1A–D). Higher magnification images also confirm that structure and pore size were maintained (Fig. S5).

Mechanical characterization of cryogels reveals that interconnected porosity and swelling ratio did not differ between empty and loaded gels, but the swelling ratio was affected by ionic crosslinking to form tough cryogels (Fig. 1F and G). Gel stiffness remained unchanged before and after ionic crosslinking (Fig. 1H). These mechanical properties are consistent with previous results [34].

IL-12 and IFN-γ are inflammatory cytokines chosen to promote M1 polarization. Cell viability and M1 polarization induced by the cytokines were confirmed using bone marrow-derived macrophages (BMDMs) (Fig. S8). BMDMs were initially polarized to M2-like macrophages via the addition of IL-13 and IL-4 cytokines. They were then either left untreated or treated for 48 h with either IFN-γ, IL-12, or a co-treatment of the two. Luminex panels confirmed that both IFN-γ treatment alone and co-treatment led to increased production of inflammatory cytokines (Fig. S9). Cell viability was improved by co-treatment with IFN-γ and IL-12 compared to IFN-γ alone (Fig. S8), so this co-treatmentwas selected for additional studies. The activity of CCL2, a known TAM chemoattractant [38], was confirmed via chemotaxis assays (Fig. S9). Based on these results, a concentration of 100 ng/cytokine/cryogel was initially chosen for in vitro studies.

The loaded gels were incubated in 1 mL 0.1% (w/v) BSA in PBS at 37 °C to evaluate cargo release. After various incubation durations, the gels were centrifuged, and the supernatant was collected and stored at −20 °C. An enzyme-linked immunosorbent assay (ELISA) revealed that the three proteins exhibited similar release curves, with approximately 90% of total cargo released after 96 h (Fig. 1I).

To evaluate the cytotoxicity of cryogels, BMDMs were treated with either control (Empty) or loaded (ILC) Gels for 48 h. No significant changes in BMDM viability for any macrophage phenotype were observed (Fig. S10).

### Examining Cryogel-Induced Repolarization in Disease-Relevant Ex Vivo Models

Bone marrow-derived macrophages (BMDMs) were generated from the femurs and tibias of FVB female mice. Cells were cultured, polarized toward an M1- or M2-like state (or left unpolarized as M0), and treated with either empty or loaded cryogels. Analysis via qRT-PCR, flow cytometry, and Luminex panels confirmed that samples treated with ILC Gels demonstrated robust activation of M1 markers and a shift toward a pro-inflammatory phenotype, consistent with the free cytokine treatment (Fig. S11). This suggests that cryogel treatment can repolarize macrophages toward M1 functions—even in cells that are pre-polarized toward M2 ex vivo.

**Fig. 2:**
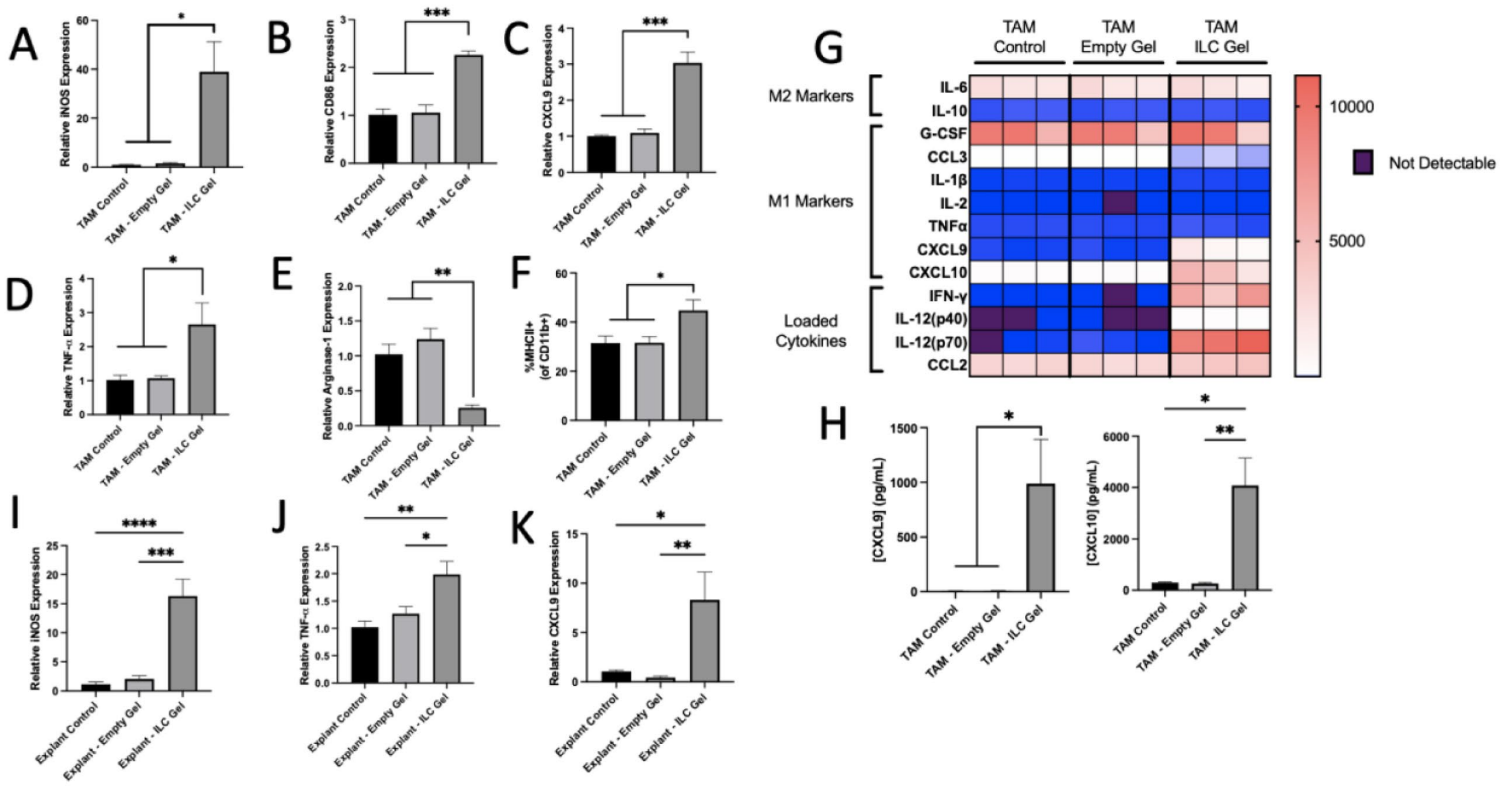
ILC Gel treatment repolarizes TAMs and explants toward an M1-like phenotype. For **A**–**H**, mammary tumors from PyMT mice were dissected and processed to single cells. CD11b cells were isolated and cultured with L929 media to select for macrophages. Loaded or empty cryogels were placed in the center of the well and the cells were treated for 48 h before being collected for qRT-PCR and flow cytometry. For **I**–**K**, mammary tumors from PyMT mice were dissected. Small (<10 mm) pieces were cut from the tumor and plated. Loaded or empty cryogels were placed next to the explant in the well and left for 48 h before the explant was collected for RT-qPCR and flow cytometry. TAMs isolated from spontaneous mammary tumors were treated for 48 h with cryogels. The ILC-treated TAMs displayed significant increases in RNA expression of iNOS, CD86, CXCL9, and TNF-α (A–D) and significantly reduced Arginase−1 expression (E). Flow cytometry analysis revealed a significant increase of MHCII receptor expression (F). Luminex analysis once again revealed large shifts toward an M1 phenotype (G), including significantly increased release of CXCL9 and CXCL10 (H). Tumor explants derived from spontaneous PyMT tumors were cultured in vitro with cryogels for 48 h. qRT-PCR analysis showed significant increases in M1 markers iNOS, TNF-α, and CXCL9 (I–K). N = 3, data are presented as mean + /− SD, *p < 0.05, **p < 0.01, ***p < 0.001, ****p < 0.0001

To model macrophage polarization in a physiologically relevant context, TAMs or tumor explants from primary mammary tumors were treated ex vivo with cryogels (Empty or ILC) for 48 h. ILC Gel-treated TAMs generated a robust phenotypic change similar to the observed BMDM responses. RNA expression of M1 markers (iNOS, CD86, CXCL9, and TNF-α) was significantly increased, while the M2 marker Arginase−1 was decreased (Fig. 2A–E). Flow cytometry indicated increased MHCII expression (Fig. 2F), and Luminex analysis identified elevated inflammatory chemokines, including CXCL9 and CXCL10 (Fig 2G and H). Tumor explants from PyMT mice were co-cultured ex vivo for 48 h with the ILC Gel. ILC Gel treatment significantly increased iNOS, TNF-α, and CXCL9 expression compared to controls, consistent with a shift toward M1 functions (Fig. 2I–K). To ensure cells would remain viable throughout the duration of the treatment, explants were also cultured for 96 h in media, and a cell viability assay confirmed the presence of live cells 4 days post-dissection (Fig. S12). These combined results suggest that ILC Gels are capable of repolarizing immunosuppressive TAMs to a more anti-tumor phenotype.

### Evaluating Efficacy of Cryogel Treatment In Vivo

Encouraging in vitro and ex vivo results justified the evaluation of potential anti-tumor effects of ILC Gels injected peritumorally. To establish an in vivo model, 1 × 10^6^ 17L3C PyMT cells were injected into the #4 mammary fat pad of FVB mice. Once tumors were palpable, an Empty Gel control or an ILC Gel (loaded with 500 ng each of IFN-γ and IL-12 as well as 100 ng of CCL2) was injected peritumorally for a 4-day treatment. As no significant changes were noted between control versus Empty Gel treatments in the in vitro and ex vivo studies, we elected to only compare empty and loaded gels, consistent with our obligation to minimize animal use. The 4-day treatment window was designed to assess the immediate impact of cryogel treatment on TAMs and other tumor-associated immune cells, rather than the capability to modulate tumor growth.

**Fig. 3:**
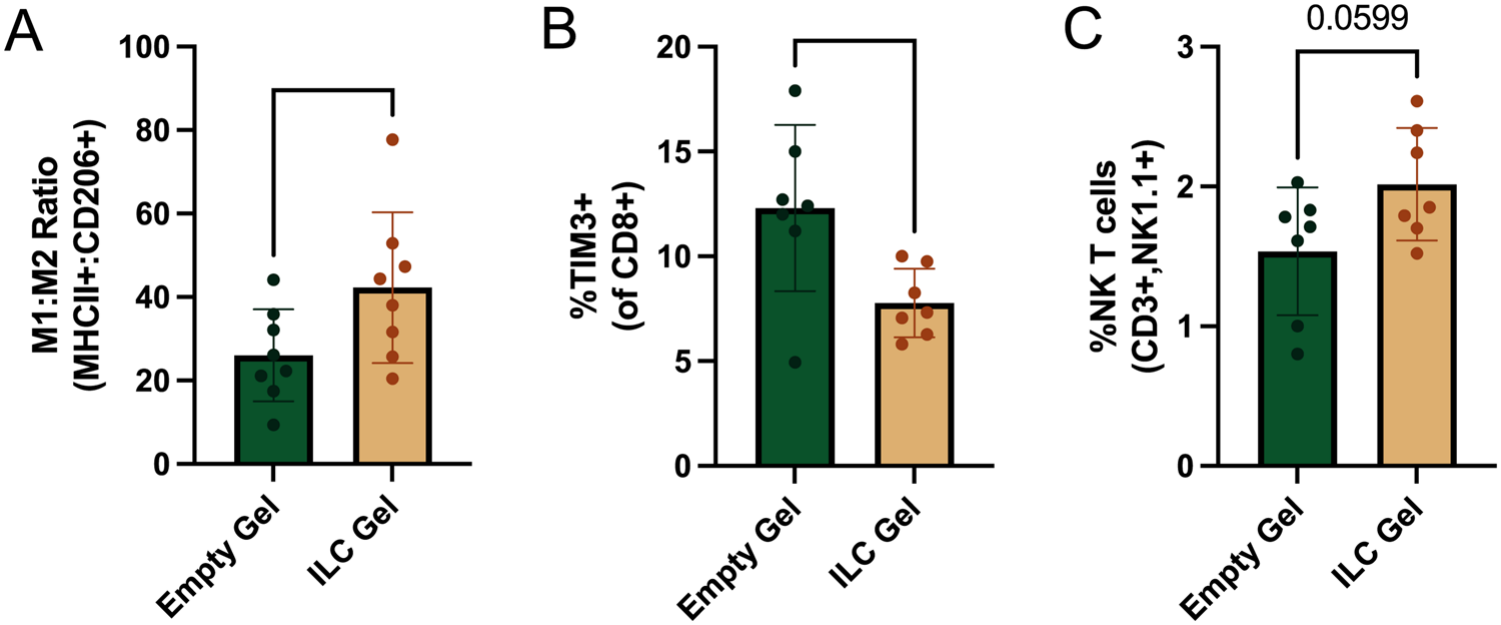
ILC Gel treatment significantly shifts macrophage polarization during 4-day treatments. FVB female mice between 8 and 12 weeks were orthotopically injected in their fourth mammary fat pad with 1x10^6^ PyMT-17L3C breast tumor cells. Tumors were given 4–5 weeks to develop. Once measurable, a single cryogel (either empty or loaded with 500 ng/cytokine (IFN-y and IL-12) and 100 ng of CCl2 was injected peritumorally. Four days post-injection, mice were euthanized, and tumors were dissected for analysis via flow cytometry. Flow cytometry revealed a significant increase in the M1:M2 macrophage ratio in the tumor. From the population of CD45 + , CD11b + /CD11c-, F4/80hi + cells, th+, CD11b+/CD11c-, F4/80hi+e ratio of MHCII + :CD206 + cells was used to determine the M1:M2 ratio (**A**). Flow cytometry also revealed a significant reduction in tumor-associated TIM3 + CD8 + T cell (**B**) as well as a strong trend in the percentage of NK T cells in treated groups (**C**). (EG and ILC n = 8, data presented as mean + /− SD, *p < 0.01). An antibody directed against B220 was used to determine the percentage of B cells in tumors. Tumors containing more than 25% B cells were removed from the analysis, as it was assumed these tumors included lymph nodes that could potentially skew results.

After 4 days of treatment, mice were euthanized, and their tumors were dissected for analysis. Flow cytometry revealed a significant increase in the M1:M2 macrophage ratio in tumors treated with the ILC Gel (Fig. 3A). TIM3 is a known exhaustion marker on T cells [46]. There was also a significant reduction in exhausted TIM3 + CD8 + T cells and a notable trend in increased NK T cells (Fig. 3B and C), suggesting that the modulation of TAM polarization also influenced other tumor-associated immune cells, including T cells.

**Fig. 4:**
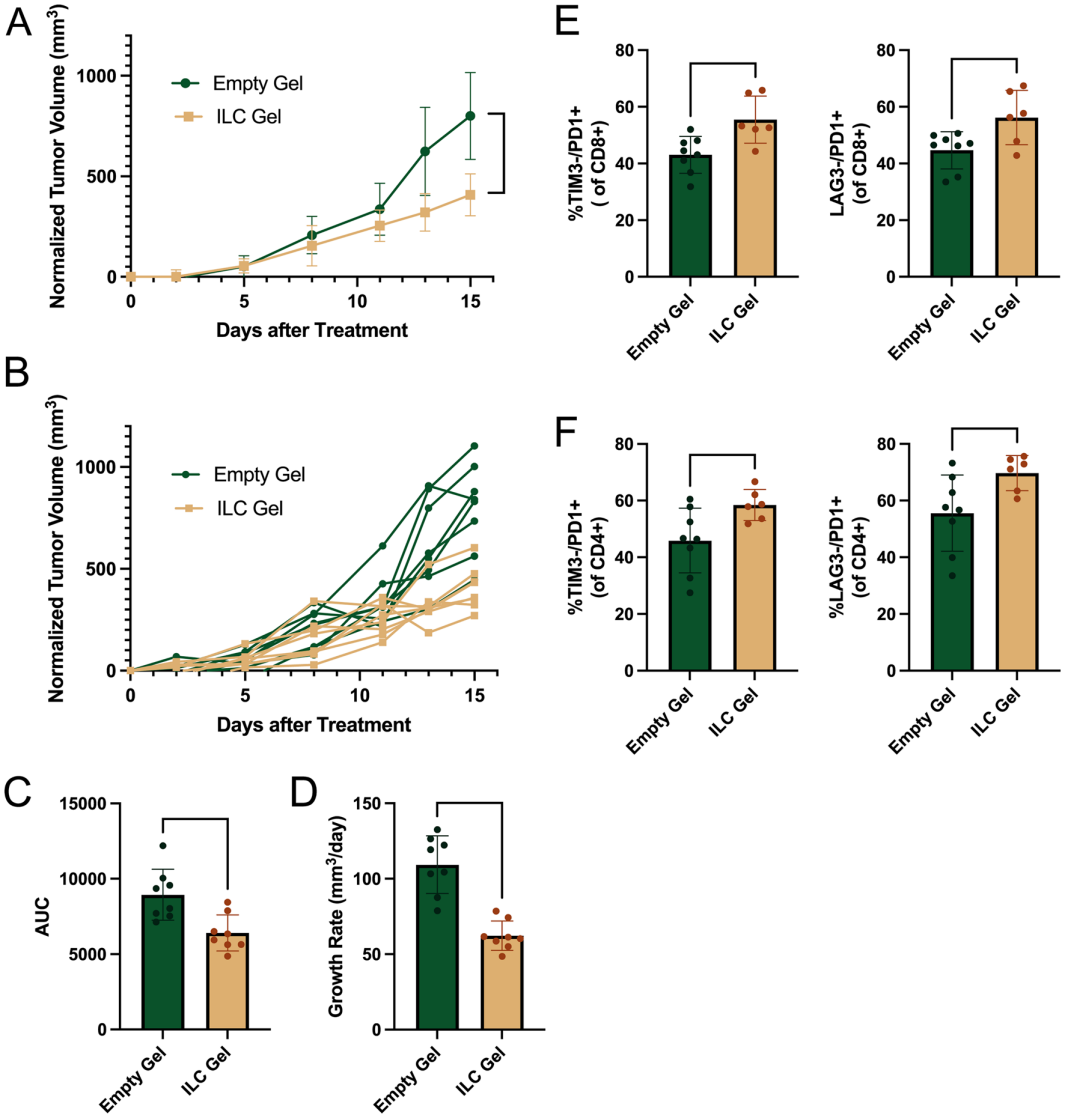
ILC Gel treatment significantly suppresses tumor progression and alters immune composition. Tumors were inoculated in female FVB mice (age 8–12 weeks) as previously described. Once measurable, a single cryogel (either empty or loaded with 500 ng/cytokine (IFN-y and IL-12) and 100 ng of CCl2 were injected peritumorally. 15 days post-injection, mice were euthanized, and tumors were dissected for analysis via flow cytometry. After 15 days of treatment, the ILC gel had significantly suppressed tumor growth (**A** and **B**) and significantly decreased the area under the curve for each growth curve (**C**), as well as significantly decreased the average daily growth (**D**). Flow cytometry found significant increases in tumor-associated TIM3-/PD1 + and LAG3-/PD1 + populations of both CD8 + and CD4 + T cells for the loaded cryogel group during the long-term study (**E** and **F)**. EG and ILC n = 8, data are presented as mean + /− SD, *p < 0.01, **p < 0.001, ***p < 0.0001. An antibody directed against B220 was used to determine the percentage of B cells in tumors. Tumors containing more than 25% B cells were removed from the analysis, as it was assumed these tumors included lymph nodes that could potentially skew results.

After establishing that the loaded cryogel rapidly altered TAM polarization, we repeated the study with a 15-day post-treatment duration to assess immune cell changes over a longer period and to evaluate the impact of injectable ILC Gels on tumor growth, allowing tumors to grow until they approached humane endpoint criteria. There were no significant changes in animal weights post-treatment and no significant changes in liver and kidney function based on serum toxicology at 15 days post-treatment (Fig. S13). Treatment with ILC Gels suppressed tumor growth compared to the Empty Gel after 15 days of therapy (Fig. 4A). The areas under the growth curve for each individual tumor and average growth rates were significantly reduced in the ILC Gel group compared to the Empty Gel group (Fig. 4B and C).

At endpoint, the tumors were surgically resected and processed for flow cytometry. Even after 15 days of treatment, significant changes in the tumor-associated T-cell population were observed, consistent with preceding TAM polarization toward M1 functions. This is unsurprising as M1-like macrophages can recruit and activate T cells [47, 48] and both IL-12 and IFN-γ can directly impact T cells [49, 50]. LAG3, in addition to TIM3, is recognized as an exhaustion marker on T cells [46]. Although PD1 expression can lead to exhaustion, it is initially seen as an activation marker [51, 52]. There was a significant increase in both CD8 + and CD4 + T cells that were TIM3-/PD1 + and LAG3-/PD1 + suggesting an increase in non-exhausted T cells (Fig. 4D and E). However, 15 days after cryogel administration, unlike the 4-day study, no significant changes in the polarization of TAMs were observed via flow cytometry or qRT-PCR.

Overall, these results suggest that localized delivery of inflammatory cytokines slows tumor development and enhances TME immunocompetence through TAM repolarization and reducing T-cell exhaustion.

**Fig. 5:**
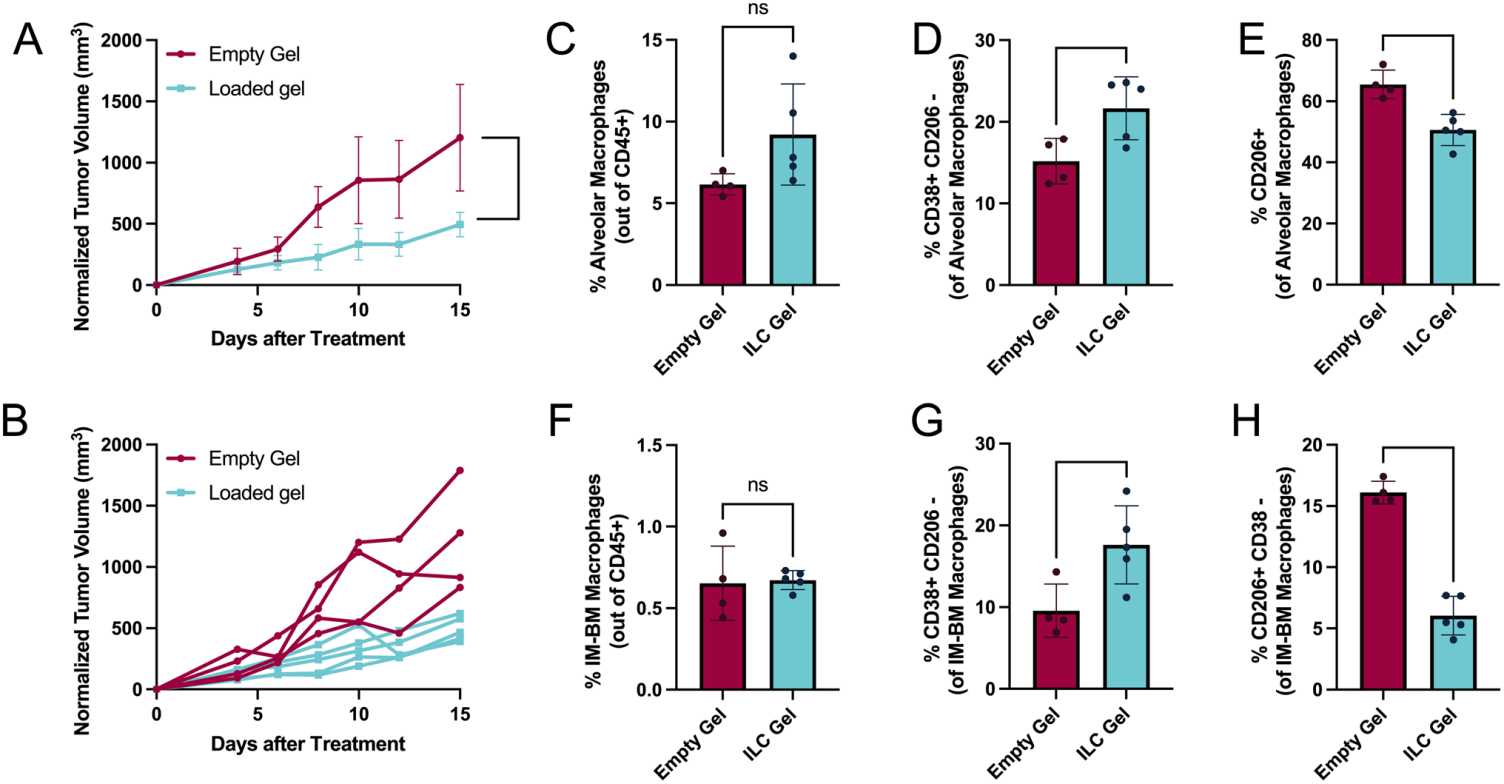
Localized multi-cryogel treatment significantly reduces primary tumor growth and shifts macrophage populations in the lungs. Tumors were inoculated in female FVB mice (age 8–12 weeks) as previously described. Once measurable, three cryogels (either empty or loaded with 500 ng/cytokine (IFN-y and IL-12) and 100 ng of CCl2 were injected peritumorally around the tumor. Fifteen days post-injection, mice were euthanized, and tumors and lungs were dissected for analysis via flow cytometry. After 15 days of treatment, the three ILC gels had significantly suppressed tumor growth as determined by the area under the curve (**A** and **B**). The multi-gel treatment did not significantly change the number of alveolar (**C**) or interstitial bone marrow-derived (IM-BM) (**F**) macrophages in the lungs, but it significantly increased M1 markers (**D**, **G**) and decreased M2 markers (**E**, **H**) for both alveolar and interstitial macrophages. (EG n = 4, ILC n = 5, data are presented as mean + /− SD, *p < 0.01, **p < 0.001, ***p < 0.0001, ****p < 0.00001)

We interpreted the outcomes data from the 15-day treatment as promising. Therefore, after assessing the impact of a single cryogel, we examined the effects of treatment using multiple cryogels to surround the tumor. Three cryogels, each loaded with 500 ng IFN-γ, IL-12, and 100 ng CCL2, were injected peritumorally approximately 120° apart to provide more spatially uniform treatment. Mice were euthanized at 15 days post-treatment for analysis. In the multi-gel experiment, tumor growth separation between the treatment and control groups was observed on Day 8 (Fig. 5A), earlier than the similar response observed on Day 13 with the single cryogel treatment (Fig. 4A). Notably, we also observed significant changes in the lung macrophages for this study (Fig. 5C–H). In both the alveolar and interstitial macrophages, the percentage of CD38 + /CD206- cells increased (Fig. 5D and G), while the percentage of CD206 + and CD206 + /CD38- cells decreased (Fig. 5E and H), suggesting a shift toward M1 polarization and away from M2 polarization within the lungs. Interestingly, the total number of macrophages for both populations did not significantly increase (Fig. 5C and F), implying that rather than creating an influx of M1 macrophages, the ILC treatment may be polarizing the resident cells in the lungs. These results demonstrate that our cryogel induces both local anti-tumor effects against the primary tumor and systemic anti-tumor effects in the lungs, an area to which breast tumors are likely to metastasize.

## Discussion

Repurposing tumor-associated macrophages (TAMs) as an anticancer immunotherapy is gaining attention due to its potential advantages over other immune cell targeting strategies [40, 45]. Recent studies have highlighted perioperative interventions using hyaluronic acid-based or bioresponsive fibrin gels to stimulate M1 macrophage polarization, enhancing the anti-tumor response [53, 54]. This TAM-centric approach addresses the limitations of T-cell-based therapies, particularly the lack of T-cell infiltration in solid tumors.

Systemic delivery of pro-inflammatory cues such as IL-12 and IFN-γ for TAM repolarization often leads to adverse effects [55–57]. Delivering these inflammatory cytokines via an injectable cryogel overcomes the barriers associated with systemic delivery. By placing the cryogel adjacent to the tumor, we hypothesize that cytokines elute locally, potentially bypassing the first-pass effect that occurs with systemically delivered drugs and allowing a higher, more bioavailable dosage of cytokine to be delivered to the tumor. Previous studies have shown that there are denser populations of macrophages around the periphery of solid tumors [37]. Thus, our expectation is that macrophages at the periphery would be the most impacted by treatment. Future studies will also look into potential cancer cell migration as a result of the induced inflammatory environment; however, there was little sign of PyMT cancer cells in the lungs of either the control or treated mice, indicating the treatment does not induce significant cancer cell migration.

While there is potential for off-target side effects with any treatment involving the delivery of inflammatory cytokines, our localized delivery is better equipped to avoid these toxicities as compared to systemic deliveries, and our current data suggest a lack of liver and kidney toxicity in treatments with a single cryogel. Future studies will be completed to confirm that only negligible and nontoxic amounts of the cytokines enter the bloodstream or adjacent tissues, and that the addition of more cryogels in a treatment dose does not induce significant toxicity.

Sustained release of cytokines from the cryogel for up to 96 h (for IFN-γ) provides a greater dose-time area under the curve for TAM repolarization relative to an intertumoral bolus or IV injection that is rapidly diluted. Additional evidence of this mechanism is offered in the comparison of faster and more robust suppression of tumor progression in mice that received three ILC Gels vs. a single ILC Gel. Presumably, the local dose of inflammatory cytokines is greater and affects a greater surface area of the tumor in mice treated with three ILC Gels, increasing the area under the curve even if the release kinetics are unchanged.

Our study aimed to demonstrate the anti-tumor potential of this treatment. This local TAM-focused immunotherapy suppressed tumor progression without significant toxicity. Initially, upon examining changes in the tumor environment following 4 days of treatment, we found significant changes in the ratio of M1:M2 macrophages, with ILC-treated TAMs shifting toward the M1 phenotype. We also observed changes in T-cell populations. To better understand the effects of the ILC Gel on the tumor immune environment and tumor growth, we repeated the single cryogel treatment with a takedown 15 days post-injection. After 15 days, the ILC Gel significantly suppressed tumor growth. Within immune cells, we observed an increase in non-exhausted CD8 + and CD4 + T cells, 15 days after a single cryogel treatment. We hypothesize that the absence of changes in macrophage populations at end point was due to the single ILC Gel treatment 15 days prior to tumor tissue analysis. Since we confirmed macrophage repolarization occurred closer to the start of treatment, the macrophages would have the potential to influence other cells but may have begun repolarizing back to M2-like functions after 15 days of exposure to the tumor microenvironment.

The ILC Gel has no known direct chemotaxis function on T cells, suggesting that T-cell infiltration occurs secondary to macrophage repolarization. While M1 macrophages are known to act in an anti-tumor manner, they can also elicit cytotoxic T-cell responses. Previous studies have also found that attempting to repolarize macrophages toward an M1-like state has led to increased T-cell presence [45]. Results from the 15-day treatment suggest that anti-tumor T-cell activity contributes to the reduction in tumor growth following ILC treatment. Changes in T-cell activation status, which include higher percentages of PD1 + T cells, suggest future studies should determine whether ILC Gels could be rationally combined with anti-PD1 checkpoint inhibitors to amplify the efficacy of treatment.

The finding that localized treatment of mammary tumors with ILC Gels induces phenotypic shifts in interstitial and alveolar macrophages in the lungs opens up additional therapeutic possibilities. A shift toward anti-tumor macrophage phenotypes in the lungs following cryogel treatment at the primary tumor site may create an inhospitable environment with the potential to reduce metastasis to the lungs. Our current model calls for a humane endpoint before robust lung metastases can form. However, future studies will incorporate tumor resection models to better assess inhibition of metastatic potential and extend the duration of the studies to examine the biological impact at longer timepoints.

While the scope of this study was to provide initial evidence of the potential for altering localized TAM polarization with locally delivered therapeutics, we hope to expand on these concepts. During these studies, we recognized the potential for an optimized approach incorporating the sequential release of chemokines (initially, for TAM chemoattraction) followed by cytokines (delayed, for reprogramming of the TAMs attracted to the cryogel). We hypothesize that making cryogel modifications to separate the release profiles of the chemokine and cytokines will further enhance TAM repolarization and anti-tumor immunity.

From a translational perspective, the cryogel platform offers promising potential due to its modularity and injectability; however, several factors require further investigation. While the cryogels used in this study were freshly synthesized, they can be lyophilized and then rehydrated months later, supporting the potential for long-term storage. Future studies will look to confirm that bioactivity and structural integrity are not compromised even after months of long-term storage in freezer conditions. Variability in tumor mechanical properties and immune microenvironments across patients may also influence treatment outcomes. To address this, future studies will test the efficacy across diverse tumor models to evaluate robustness and inform patient-specific therapeutic strategies.

The cryogels used in our studies were meticulously designed to allow for injectability while maintaining a desirable macroporous structure, akin to a synthetic ‘lymph node,’ as successfully demonstrated in prior research [34]. While our choice to administer the cryogel peritumorally aimed to assess the modulation of TAMs within the tumor microenvironment, it is notable that this same approach can complement the current standard of surgical excision, as demonstrated by Park et al. using a different hydrogel system [53]. Thus, a cryogel can be effectively implanted into the resection site, aligning with existing treatment protocols, and potentially enhancing clinical relevance. The rationale for such an approach includes the immune-eradication of residual tumor cells in the resection site that would not otherwise occur and may contribute to metastatic spread, which is responsible for the majority of deaths from breast cancer. Overall, our findings provide evidence that peritumoral injection of a TAM repolarization depot reduces the immunosuppressive tumor microenvironment, shifts macrophage phenotypes, and supports a systemic anti-tumor immune response.

## Supplementary Information

Below is the link to the electronic supplementary material.

Supplementary file1 (PDF 25,465 KB)

## Data Availability

Data are available from the corresponding author upon reasonable request.

## References

[1] U.S. Breast Cancer Statistics | Breastcancer.org. Accessed: Oct. 02, 2021. https://www.breastcancer.org/symptoms/understand_bc/statistics?gclid=CjwKCAjwqeWKBhBFEiwABo_XBnHSwNc53kIm3LmQjP9PjjSRWFFZ1AQG9gVMfniiyOgFoMjGaBzufBoCcm8QAvD_BwE

[2] Siegel, R. L., K. D. Miller, H. E. Fuchs, and A. Jemal. Cancer statistics, 2022. *CA Cancer J Clin*. 72(1):7–33, 2022. 10.3322/CAAC.21708. 35020204 10.3322/caac.21708

[3] Chowdhury, P. S., K. Chamoto, and T. Honjo. Combination therapy strategies for improving PD-1 blockade efficacy: a new era in cancer immunotherapy. *J Intern Med*. 283(2):110–120, 2018. 10.1111/joim.12708. 29071761 10.1111/joim.12708

[4] Yang, Y. Cancer immunotherapy: harnessing the immune system to battle cancer. *J Clin Investig*. 125(9):3335–3337, 2015. 10.1172/JCI83871.It. 26325031 10.1172/JCI83871 PMC4588312

[5] Farkona, S., E. P. Diamandis, and I. M. Blasutig. Cancer immunotherapy: the beginning of the end of cancer? *BMC Med*. 14(73):1–18, 2016. 10.1186/s12916-016-0623-5. 27151159 10.1186/s12916-016-0623-5 PMC4858828

[6] Carretero-González, A., et al. Analysis of response rate with ANTI-PD1/PD-L1 monoclonal antibodies in advanced solid tumors: a meta-analysis of randomized clinical trials. *Oncotarget*. 9(9):8706–8715, 2018. 10.18632/oncotarget.24283. 29492229 10.18632/oncotarget.24283 PMC5823578

[7] Nishino, M., N. H. Ramaiya, H. Hatabu, and F. S. Hodi. Monitoring immune-checkpoint blockade: response evaluation and biomarker development. *Nat Rev Clin Oncol*. 14(11):655–668, 2017. 10.1016/j.physbeh.2017.03.040. 28653677 10.1038/nrclinonc.2017.88 PMC5650537

[8] Hou, B., Y. Tang, W. Li, Q. Zeng, and D. Chang. Efficiency of CAR-T therapy for treatment of solid tumor in clinical trials: a meta-analysis. *Dis Markers*. 2019. 10.1155/2019/3425291. 30886654 10.1155/2019/3425291 PMC6388318

[9] Rabinovich, G. A., D. Gabrilovich, and E. M. Sotomayor. Immunosuppressive strategies that are mediated by tumor cells. *Annu Rev Immunol*. 25:267–296, 2007. 10.1146/annurev.immunol.25.022106.141609.IMMUNOSUPPRESSIVE. 17134371 10.1146/annurev.immunol.25.022106.141609 PMC2895922

[10] Tormoen, G. W., M. R. Crittenden, and M. J. Gough. Role of the immunosuppressive microenvironment in immunotherapy. *Adv Radiat Oncol*. 3(4):520–526, 2018. 10.1016/j.adro.2018.08.018. 30370351 10.1016/j.adro.2018.08.018 PMC6200899

[11] Condeelis, J., and J. W. Pollard. Macrophages: obligate partners for tumor cell migration, invasion, and metastasis. *Cell*. 124(2):263–266, 2006. 10.1016/j.cell.2006.01.007. 16439202 10.1016/j.cell.2006.01.007

[12] Qian, B. Z., and J. W. Pollard. Macrophage diversity enhances tumor progression and metastasis. *Cell*. 141(1):39–51, 2010. 10.1016/j.cell.2010.03.014. 20371344 10.1016/j.cell.2010.03.014 PMC4994190

[13] Noy, R., and J. W. Pollard. Tumor-associated macrophages: from mechanisms to therapy. *Immunity*. 41(1):49–61, 2014. 10.1016/j.immuni.2014.06.010. 25035953 10.1016/j.immuni.2014.06.010 PMC4137410

[14] Mills, C. D., K. Kincaid, J. M. Alt, M. J. Heilman, and A. M. Hill. M-1/M-2 Macrophages and the Th1/Th2 Paradigm. *J Immunol*. 164(12):6166–6173, 2000. 10.4049/jimmunol.164.12.6166. 10843666 10.4049/jimmunol.164.12.6166

[15] Ley, K. M1 means kill; M2 means heal. *J Immunol*. 199(7):2191–2193, 2017. 10.4049/jimmunol.1701135. 28923980 10.4049/jimmunol.1701135

[16] Oshi, M., et al. M1 macrophage and M1/M2 ratio defined by transcriptomic signatures resemble only part of their conventional clinical characteristics in breast cancer. *Sci Rep*. 10(1):1–12, 2020. 10.1038/s41598-020-73624-w. 33024179 10.1038/s41598-020-73624-w PMC7538579

[17] Jayasingam, S. D., M. Citartan, T. H. Thang, A. A. Mat Zin, K. C. Ang, and E. S. Ch’ng. Evaluating the polarization of tumor-associated macrophages into M1 and M2 phenotypes in human cancer tissue: technicalities and challenges in routine clinical practice. *Front Oncol*. 9:1–9, 2020. 10.3389/fonc.2019.01512. 10.3389/fonc.2019.01512 PMC6992653 32039007

[18] Singhal, S., et al. Human tumor-associated monocytes/macrophages and their regulation of T cell responses in early-stage lung cancer. *Sci Transl Med*. 11:479, 2019. 10.1126/scitranslmed.aat1500. 10.1126/scitranslmed.aat1500 PMC6800123 30760579

[19] Cassetta, L., et al. Human tumor-associated macrophage and monocyte transcriptional landscapes reveal cancer-specific reprogramming, biomarkers, and therapeutic targets. *Cancer Cell*. 35(4):588–602, 2019. 10.1016/j.ccell.2019.02.009. 30930117 10.1016/j.ccell.2019.02.009 PMC6472943

[20] Henze, A., and M. Mazzone. The impact of hypoxia on tumor-associated macrophages. *J Clin Invest*. 126(10):3672–3679, 2016. 10.1172/JCI84427.marrow. 27482883 10.1172/JCI84427 PMC5096805

[21] Duan, Z., and Y. Luo. Targeting macrophages in cancer immunotherapy. *Signal Transduct Target Ther*. 6(1):1–21, 2021. 10.1038/s41392-021-00506-6. 33767177 10.1038/s41392-021-00506-6 PMC7994399

[22] Fang, Z., et al. Myeloid-derived suppressor cell and macrophage exert distinct angiogenic and immunosuppressive effects in breast cancer. *Oncotarget*. 8(33):54173, 2017. 10.18632/ONCOTARGET.17013. 28903332 10.18632/oncotarget.17013 PMC5589571

[23] Huang, Y., et al. Vascular normalizing doses of antiangiogenic treatment reprogram the immunosuppressive tumor microenvironment and enhance immunotherapy. *Proc Natl Acad Sci USA*. 109(43):17561–17566, 2012. 10.1073/PNAS.1215397109/SUPPL_FILE/PNAS.201215397SI.PDF. 23045683 10.1073/pnas.1215397109 PMC3491458

[24] Duluc, D., et al. Interferon-γ reverses the immunosuppressive and protumoral properties and prevents the generation of human tumor-associated macrophages. *Int J Cancer*. 125(2):367–373, 2009. 10.1002/ijc.24401. 19378341 10.1002/ijc.24401

[25] Kerneur, C., C. E. Cano, and D. Olive. Major pathways involved in macrophage polarization in cancer. *Front Immunol*. 13:1026954, 2022. 10.3389/FIMMU.2022.1026954/BIBTEX. 36325334 10.3389/fimmu.2022.1026954 PMC9618889

[26] Watkins, S. K., N. K. Egilmez, J. Suttles, and R. D. Stout. IL-12 rapidly alters the functional profile of tumor-associated and tumor-infiltrating macrophages in vitro and in vivo. *J Immunol*. 178(3):1357–1362, 2007. 10.4049/JIMMUNOL.178.3.1357. 17237382 10.4049/jimmunol.178.3.1357

[27] Wang, N., H. Liang, and K. Zen. Molecular mechanisms that influence the macrophage M1–M2 polarization balance. *Front Immunol*. 5:113523, 2014. 10.3389/FIMMU.2014.00614/BIBTEX. 10.3389/fimmu.2014.00614 PMC4246889 25506346

[28] Oliver, B., E. Jackson, and H. Soliman. Immunostimulators and Immunomodulators in Cancer Treatment. *Novel Approaches and Strategies for Biologics, Vaccines and Cancer Therapies.* 2015. 10.1016/B978-0-12-416603-5.00017-1.

[29] Miller, C. H. T., S. G. Maher, and H. A. Young. Clinical use of interferon-γ. *Ann NY Acad Sci*. 1182:69, 2009. 10.1111/J.1749-6632.2009.05069.X. 20074276 10.1111/j.1749-6632.2009.05069.x PMC6574079

[30] Leach, D. G., S. Young, and J. D. Hartgerink. Advances in immunotherapy delivery from implantable and injectable biomaterials. *Acta Biomater*. 88:15–31, 2019. 10.1016/j.actbio.2019.02.016. 30771535 10.1016/j.actbio.2019.02.016 PMC6632081

[31] Li, J., et al. Advances of injectable hydrogel-based scaffolds for cartilage regeneration. *Regen Biomater*. 6(3):129–140, 2019. 10.1093/rb/rbz022. 31198581 10.1093/rb/rbz022 PMC6547311

[32] d’Aquino, A. I., et al. Use of a biomimetic hydrogel depot technology for sustained delivery of GLP-1 receptor agonists reduces burden of diabetes management. *Cell Rep Med*. 4(11):101292, 2023. 10.1016/J.XCRM.2023.101292. 37992687 10.1016/j.xcrm.2023.101292 PMC10694761

[33] Bencherif, S. A., et al. Injectable preformed scaffolds with shape-memory properties. *Proc Natl Acad Sci*. 109(48):19590–19595, 2012. 10.1073/pnas.1211516109. 23150549 10.1073/pnas.1211516109 PMC3511752

[34] Shih, T.-Y., et al. Injectable, tough alginate cryogels as cancer vaccines. *Adv Healthc Mater*. 7(10):1701469, 2018. 10.1002/ADHM.201701469. 10.1002/adhm.201701469 PMC6467206 29441705

[35] Pérez-Luna, V. H., and O. González-Reynoso. Encapsulation of biological agents in hydrogels for therapeutic applications. *Gels*. 2018. 10.3390/GELS4030061. 30674837 10.3390/gels4030061 PMC6209244

[36] Bencherif, S. A., et al. Injectable cryogel-based whole cell cancer vaccines. *Nat Commun*. 6:6072–6078, 2016. 10.1002/cncr.27633.Percutaneous. 10.1038/ncomms8556 PMC4763944 26265369

[37] Lewis, C. E., and J. W. Pollard. Distinct role of macrophages in different tumor microenvironments. *Cancer Res*. 66(2):605–612, 2006. 10.1158/0008-5472.CAN-05-4005. 16423985 10.1158/0008-5472.CAN-05-4005

[38] Chen, Y., Y. Song, W. Du, L. Gong, H. Chang, and Z. Zou. Tumor-associated macrophages: an accomplice in solid tumor progression. *J Biomed Sci*. 26(1):78, 2019. 10.1186/S12929-019-0568-Z. 31629410 10.1186/s12929-019-0568-z PMC6800990

[39] Martin, M. D., et al. Effect of ablation or inhibition of stromal matrix metalloproteinase-9 on lung metastasis in a breast cancer model is dependent on genetic background. *Cancer Res*. 68(15):6251, 2008. 10.1158/0008-5472.CAN-08-0537. 18676849 10.1158/0008-5472.CAN-08-0537 PMC2789265

[40] Glass, E. B., et al. Optimizing mannose “click” conjugation to polymeric nanoparticles for targeted siRNA delivery to human and murine macrophages. *ACS Omega*. 4(16):16756–16767, 2019. 10.1021/acsomega.9b01465. 31646220 10.1021/acsomega.9b01465 PMC6796989

[41] Schindelin, J., et al. Fiji: an open-source platform for biological-image analysis. *Nat Methods*. 9(7):676–682, 2012. 10.1038/nmeth.2019. 22743772 10.1038/nmeth.2019 PMC3855844

[42] Ahearne, M., Y. Yang, A. J. El Haj, K. Y. Then, and K. K. Liu. Characterizing the viscoelastic properties of thin hydrogel-based constructs for tissue engineering applications. *J R Soc Interface*. 2(5):455–463, 2005. 10.1098/rsif.2005.0065. 16849205 10.1098/rsif.2005.0065 PMC1618501

[43] Tomasova, L., Z. Guttenberg, B. Hoffmann, and R. Merkel. Advanced 2D/3D cell migration assay for faster evaluation of chemotaxis of slow-moving cells. *PLoS One*. 14(7):e0219708, 2019. 10.1371/JOURNAL.PONE.0219708. 31314801 10.1371/journal.pone.0219708 PMC6636736

[44] Glass, E. B., et al. Stimulating TAM-mediated anti-tumor immunity with mannose-decorated nanoparticles in ovarian cancer. *BMC Cancer*. 22(497):1–19, 2022. 10.1186/s12885-022-09612-2. 35513776 10.1186/s12885-022-09612-2 PMC9074180

[45] Hoover, A. A., et al. Increased canonical NF-kappaB signaling specifically in macrophages is sufficient to limit tumor progression in syngeneic murine models of ovarian cancer. *BMC Cancer*. 20(1):970, 2020. 10.1186/S12885-020-07450-8. 33028251 10.1186/s12885-020-07450-8 PMC7542116

[46] Wherry, E. J., and M. Kurachi. Molecular and cellular insights into T cell exhaustion. *Nat Rev Immunol*. 15(8):486–499, 2015. 10.1038/nri3862. 26205583 10.1038/nri3862 PMC4889009

[47] Mills, C. D., and K. Ley. M1 and M2 macrophages: the chicken and the egg of immunity. *J Innate Immun*. 6(6):716, 2014. 10.1159/000364945. 25138714 10.1159/000364945 PMC4429858

[48] Duan, Z., and Y. Luo. Targeting macrophages in cancer immunotherapy. *Signal Transduction Targeted Ther*. 6(1):1–21, 2021. 10.1038/s41392-021-00506-6. 10.1038/s41392-021-00506-6 PMC7994399 33767177

[49] Tugues, S., et al. New insights into IL-12-mediated tumor suppression. *Cell Death Differentiation*. 22(2):237–246, 2014. 10.1038/cdd.2014.134. 25190142 10.1038/cdd.2014.134 PMC4291488

[50] Jorgovanovic, D., M. Song, L. Wang, and Y. Zhang. Roles of IFN-γ in tumor progression and regression: a review. *Biomarker Res*. 8(1):1–16, 2020. 10.1186/S40364-020-00228-X. 10.1186/s40364-020-00228-x PMC7526126 33005420

[51] Jubel, J. M., Z. R. Barbati, C. Burger, D. C. Wirtz, and F. A. Schildberg. The role of PD-1 in acute and chronic infection. *Front Immunol*. 11:524474, 2020. 10.3389/FIMMU.2020.00487/BIBTEX. 10.3389/fimmu.2020.00487 PMC7105608 32265932

[52] Simon, S., and N. Labarriere. PD-1 expression on tumor-specific T cells: friend or foe for immunotherapy?’. *Oncoimmunology*. 2018. 10.1080/2162402X.2017.1364828. 10.1080/2162402X.2017.1364828 PMC5739549 29296515

[53] Park, C. G., C. A. Hartl, D. Schmid, E. M. Carmona, H.-J. Kim, and M. S. Goldberg. Extended release of perioperative immunotherapy prevents tumor recurrence and eliminates metastases. *Sci Transl Med*. 10(433):1–14, 2018. 10.1126/scitranslmed.aar1916. 10.1126/scitranslmed.aar1916 29563317

[54] Chen, Q., et al. In situ sprayed bioresponsive immunotherapeutic gel for post-surgical cancer treatment. *Nat Nanotechnol*. 14(1):89–97, 2019. 10.1038/s41565-018-0319-4. 30531990 10.1038/s41565-018-0319-4

[55] Dong, C., et al. Interleukin-12 delivery strategies and advances in tumor immunotherapy. *Curr Iss Mol Biol*. 46(10):11548–11579, 2024. 10.3390/CIMB46100686. 10.3390/cimb46100686 PMC11506767 39451566

[56] Kurzrock, R., et al. Pharmacokinetics, single-dose tolerance, and biological activity of recombinant gamma-interferon in cancer patients. *Oncology (Switzerland)*. 42:41–50, 1985. 10.1159/000226083. 3921249

[57] Sriskandan, K., et al. A toxicity study of recombinant interferon-gamma given by intravenous infusion to patients with advanced cancer. *Cancer Chemother Pharmacol*. 18(1):63–68, 1986. 10.1007/BF00253067/METRICS. 3093108 10.1007/BF00253067

