# Supplementary material for: Locally Reprogramming Tumor-Associated Macrophages with Cytokine-Loaded Injectable Cryogels for Breast Cancer
Source: Ann Biomed Eng. 2025 Aug 29;53(11):3081–95. doi: 10.1007/s10439-025-03823-x (PMC12575589; doi:10.1007/s10439-025-03823-x)
Supplement: Supplementary file 1 — Supplementary file1 (PDF 25,465 KB) [file 10439_2025_3823_MOESM1_ESM.pdf]

Annals of Biomedical Engineering

Supplemental Materials

Locally Reprogramming Tumor-Associated Macrophages with Cytokine-loaded Injectable Cryogels for Breast Cancer

Sydney R. Henriques*[1], Evan B. Glass*[1], Kristen L. Hoek[2], Ori Z. Chalom[1], Abigail E. Manning[1], Sohini Roy[3,4], Diana K. Graves[5], Sarah M. Goldstein[1], Benjamin C. Hacker[6], Renjie Jin[2], Marjan Rafat[1,6], Paula J. Hurley[7,8], Laura C. Kennedy[7,8], Young J. Kim[3,9], Andrew J. Wilson[8,10], Fiona E. Yull[2,3,4,11], and Todd D. Giorgio[1,6,8,11]

***co-first author**

[1] Department of Biomedical Engineering, Vanderbilt University

[2] Department of Pharmacology, Vanderbilt University

[3] Department of Otolaryngology, Vanderbilt University Medical Center

[4] Johnson and Johnson

[5] Department of Pathology, Microbiology and Immunology, Vanderbilt University

[6] Department of Chemical and Biomolecular Engineering, Vanderbilt University

[7] Department of Medicine, Vanderbilt University Medical Center

[8] Vanderbilt Ingram Cancer Center

[9] Regeneron Pharmaceuticals

[10] Department of OB/GYN, Vanderbilt University Medical Center

[11] Program in Cancer Biology, Vanderbilt University

Corresponding author email: todd.d.giorgio@vanderbilt.edu

Supplemental Figure 1: Schematic of Cryogel Fabrication

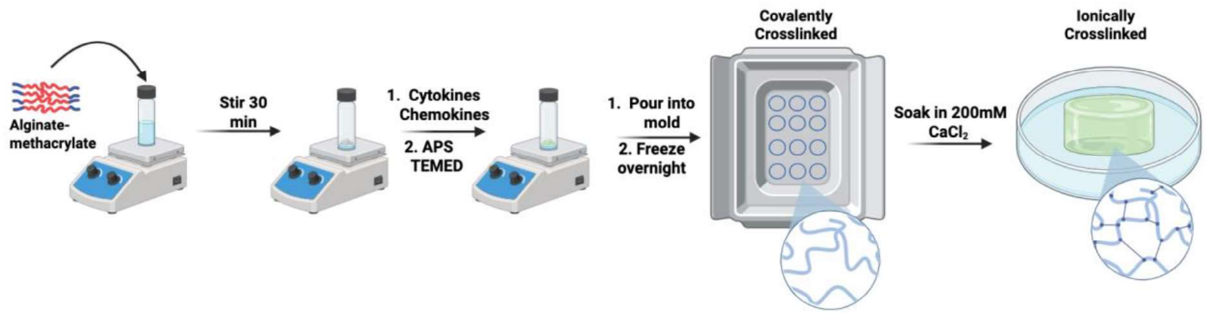

Supplemental Figure S1: Schematic of cryogel fabrication method. The cryogel is prepared by adding alginate methacrylate to DI H_20 at 1.5% w/v and allowing it to stir for ~30 min. CCL2, IL-12, and IFN-y are added before radical initiators ammonium persulfate and N,N,N′,N′-Tetramethylethane-1,2-diamine are added at 0.25% and 0.5% w/v, respectively. The cryogel solution is immediately poured into a mold and frozen overnight. The cryogels are then ionically crosslinked in 200mM $CaCl_2$ for 10 min. Cryogels are either immediately used or frozen and lyophilized for future use. Created with BioRender.com.

Supplemental Figure S2: Experimental timeline of *in vivo* mouse mammary orthotopic studies

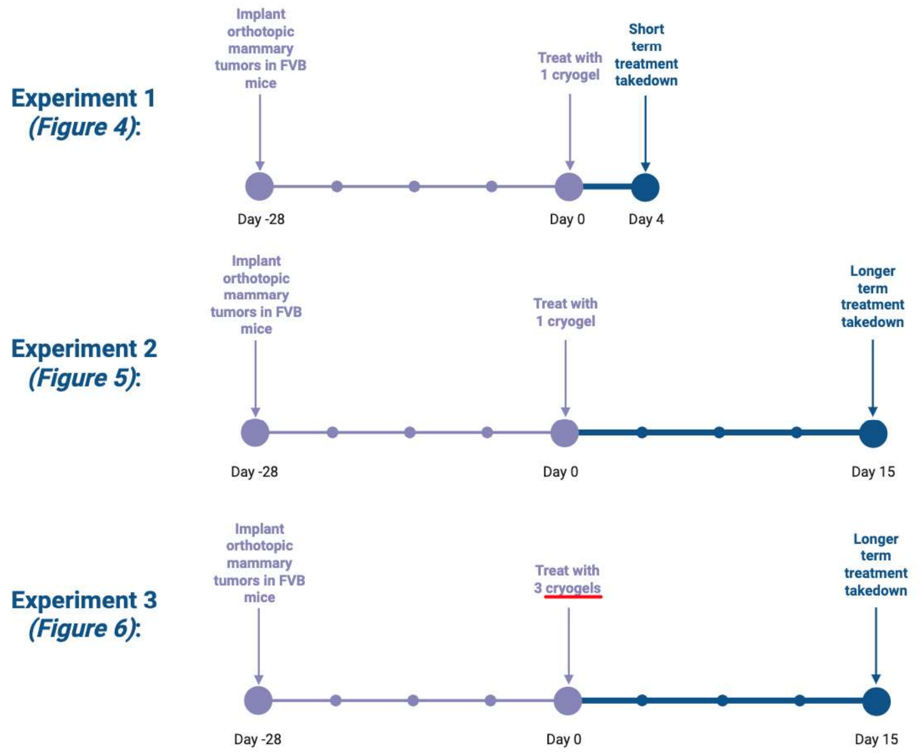

Supplemental Figure S2: Detailed timelines of the *in vivo* mammary tumor experiments performed. All *in vivo* experiments followed the same conditions other than the duration of treatment (4 or 15 days) and the number of cryogels injected (1 or 3 cryogels). Created with BioRender.com.

Supplemental Figure S3: *In vitro* Macrophage Flow Gating Strategy

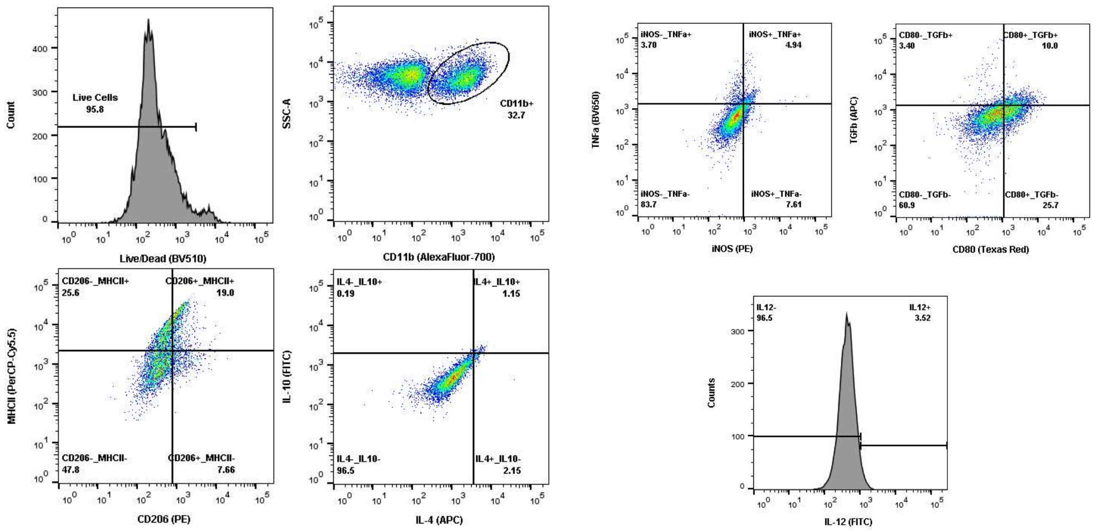

Supplemental Figure S3: Flow gating for BMDMs illustrates gating for live cells, macrophages (CD11b+), and M1 (MHCII+/CD206+ or iNOS+/TNFα+) vs M2 (MHCII-/CD206+ or IL-4+/IL-10+). Single cells were gated by excluding doublets before live cell gating occurred.

Supplemental Figure S4: ***In vivo*** **Myeloid Panels**

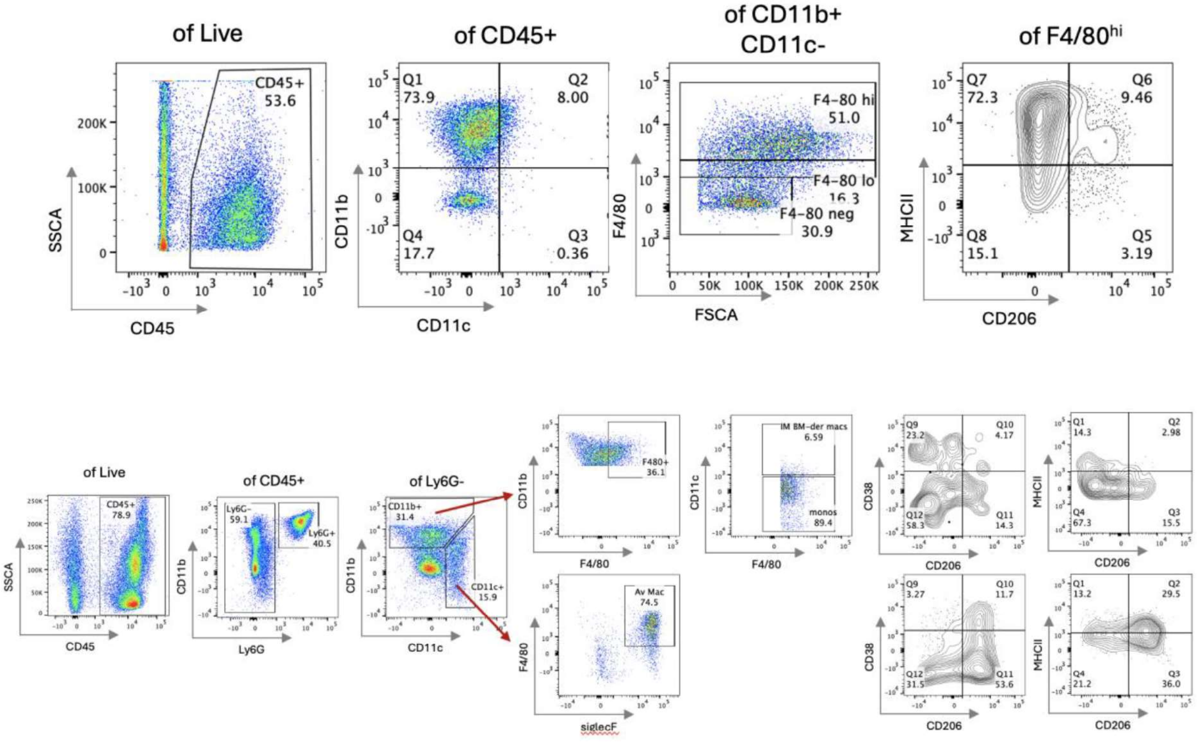

Supplemental Figure S4: Top: Flow gating strategy for myeloid tumor cells. Illustrates gating for immune cells (CD45+), monocytes (CD11b+/CD11c-), macrophages (F4/80hi), and M1/M2 (MHCII/CD206). Bottom panel: Flow gating strategy for myeloid lung cells. Illustrates gating for immune cells (CD45+), neutrophils (Ly6G+), alveolar macrophages (CD11c+/F4/80+/siglecF+), interstitial macrophages (CD11b+/CD11c+/F4/80+), M1 phenotype (CD38 and MHC II) and M2 phenotype (CD206).

Supplemental Figure S5: ***In vivo*** **T cell Flow Gating Strategies**

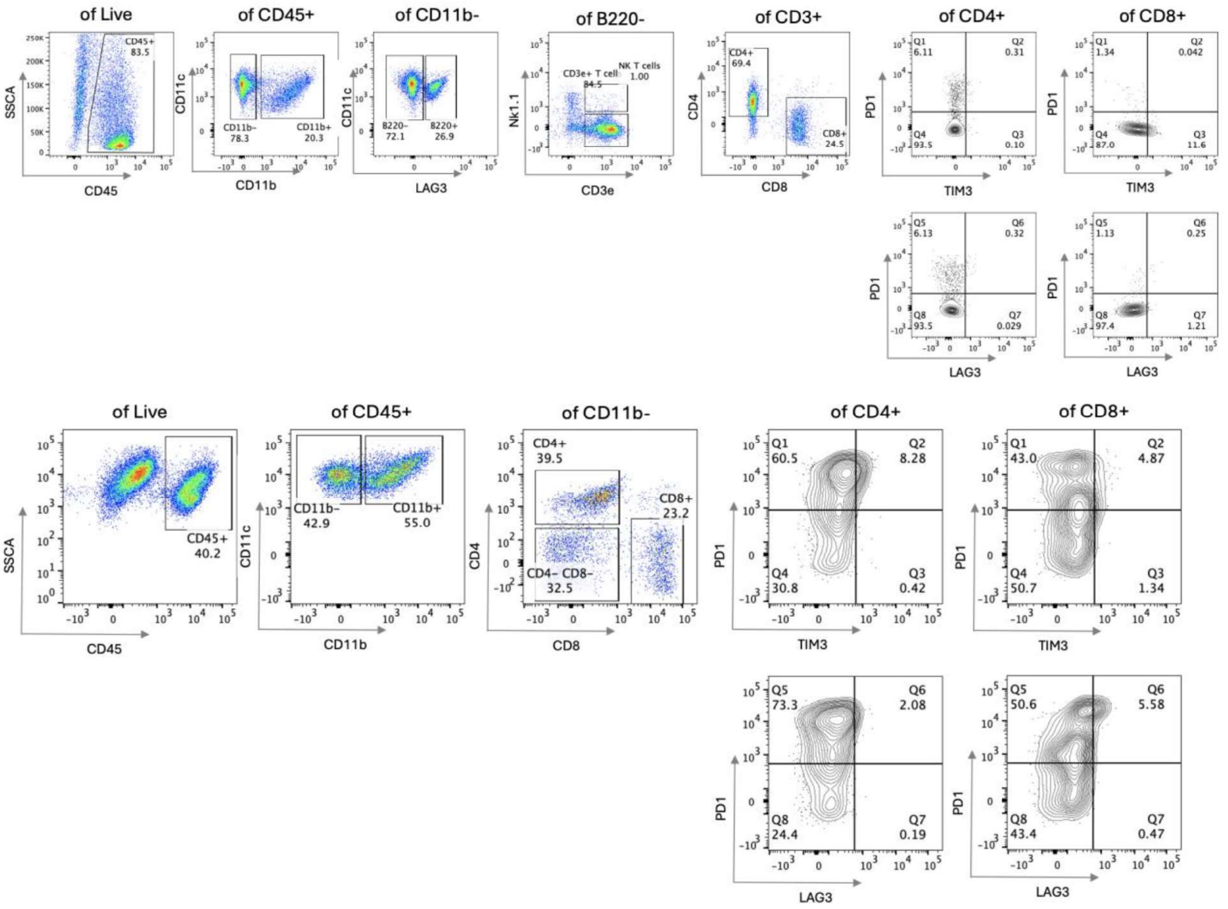

Supplemental Figure S5: Top: Initial flow gating strategy for 15 day treatment. Illustrates gating for immune cells (CD45+), lymphocytes (CD11b-), CD4+, CD8+, and exhausted T cells (PD1, TIM3, and LAG3). Bottom: Flow gating strategy for 4-day treatment, which added B cell and T cell specific markers. Illustrates gating for immune cells (CD45+), lymphocytes (CD11b-), B cells (B220+), NK T cells (NK1.1/CD3e), CD4+, CD8+, and exhausted T cells (PD1, TIM3, and LAG3).

Supplemental Figure S6: NMR Characterization of Alginate Methacrylation and Cryogelation

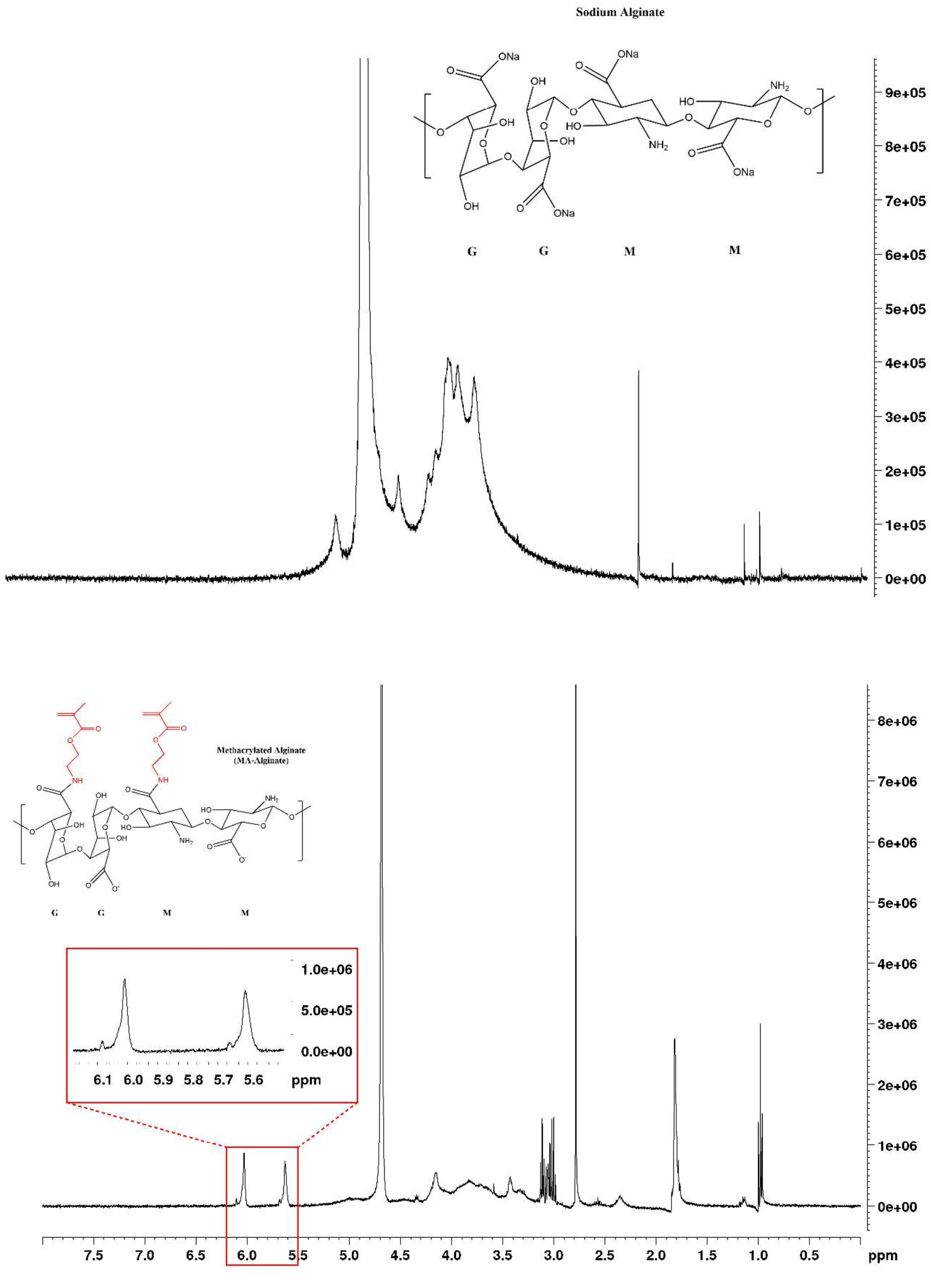

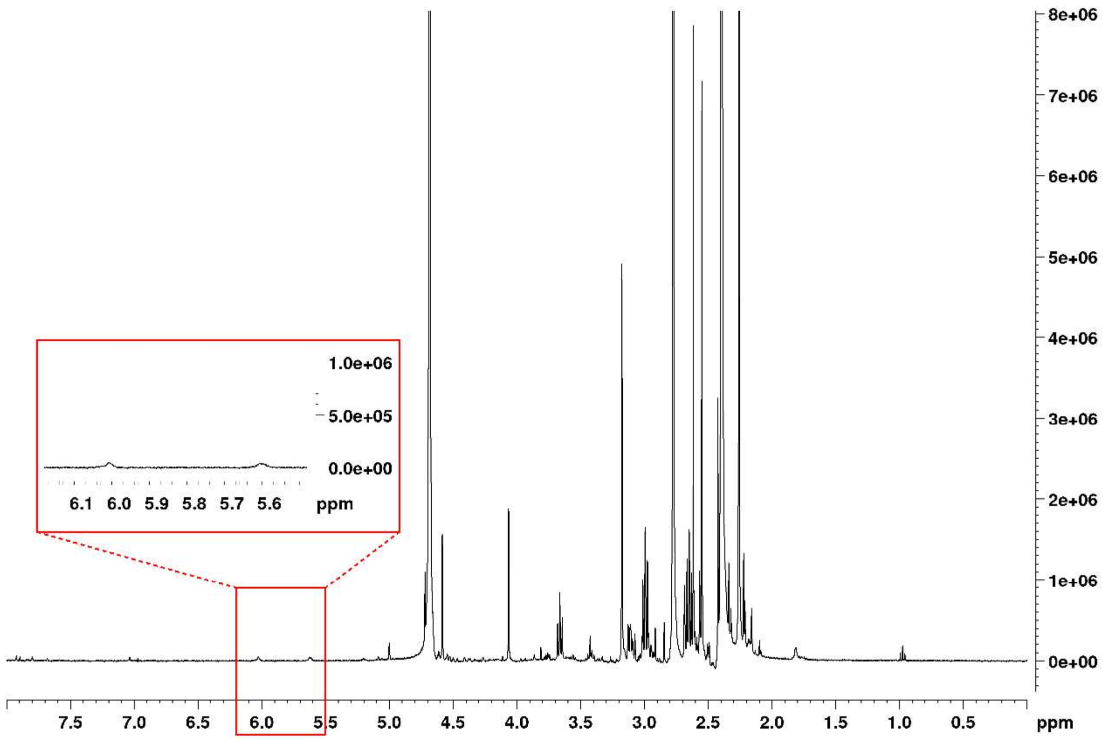

Supplemental Figure S6: NMR analysis reveals appearance of methacrylate peaks after alginate methacrylation (red box, second figure) as well as disappearance of these peaks after crosslinking during cryogelation (red box, third figure).

Supplemental Figure S7: High Magnification Cryogel SEM images

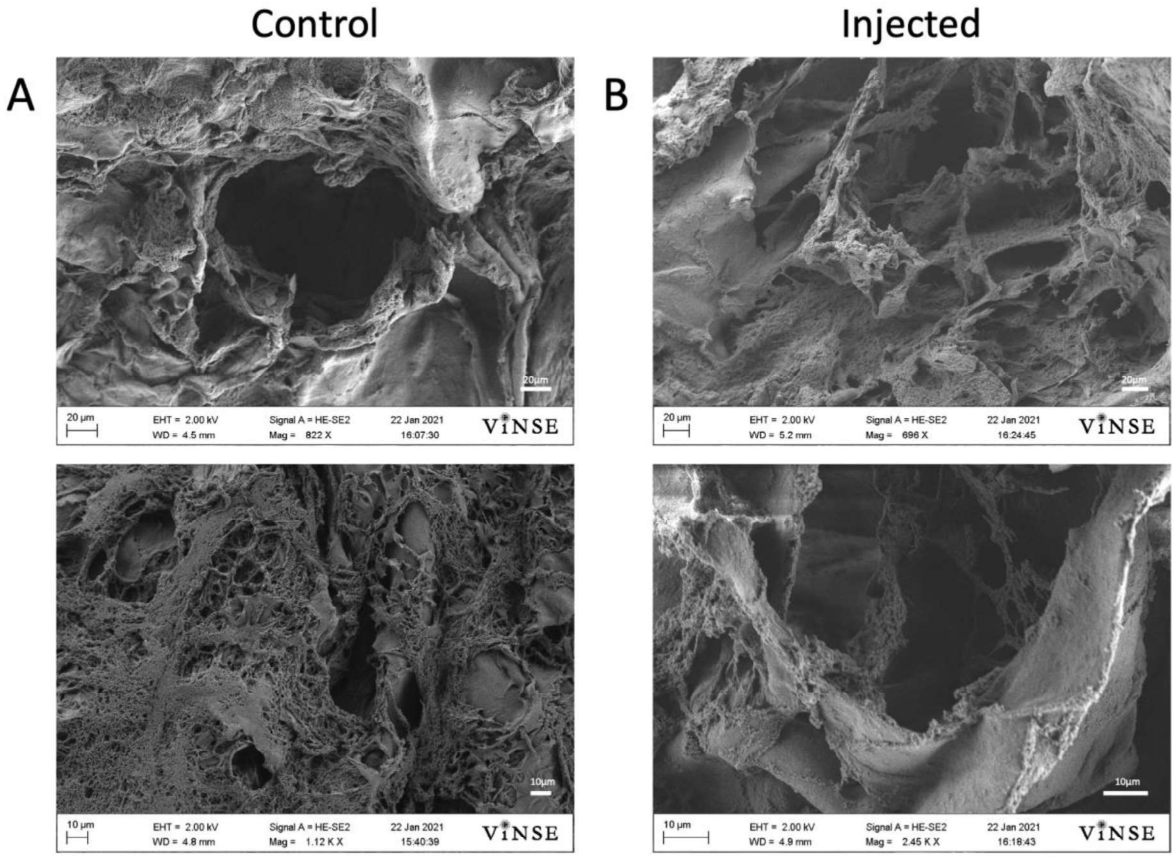

Supplemental Figure S7: Scanning electron microscropy images of both (**A**) control (non-injected) and (**B**) injected cryogels at high magnification. These images show that the porosity and structure remains unchanged post-injection through a 16G syringe.

Supplemental Figure S8: BMDM Cell Viability

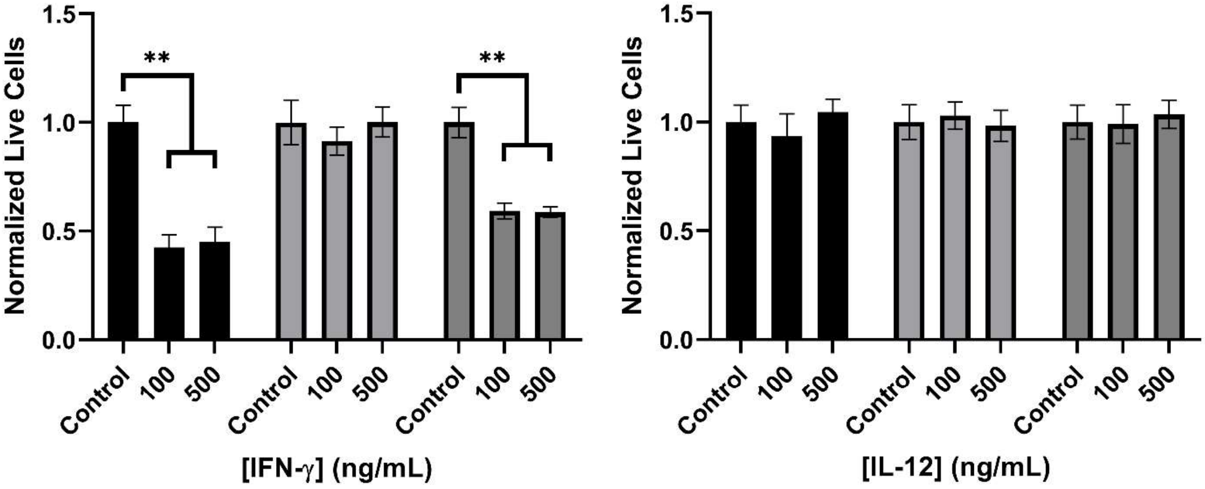

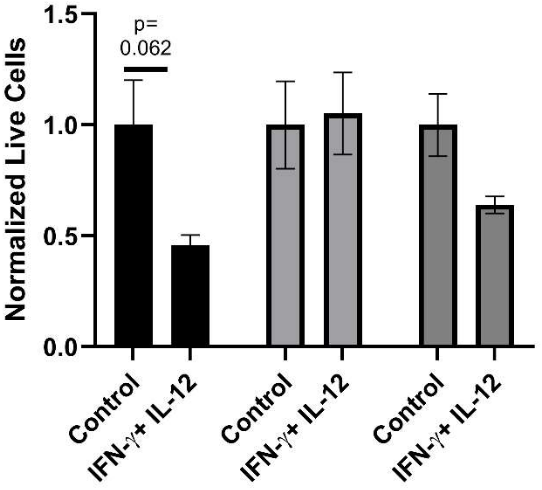

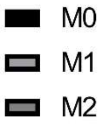

Supplemental Figure S8: BMDMs treated with free cytokines revealed a significant decrease in viability for M0 and M2 BMDMs treated with IFN-γ. However, no macrophage phenotype exhibited significant toxicity in treatments with IL-12 or a cotreatment of IFN-γ + IL-12. N=4, data presented as mean +/- SD, *$p<0.05$, **$p<0.01$.

Supplemental Figure S9: Inflammatory cytokines polarize BMDMs and chemokines induce macrophage chemotaxis.

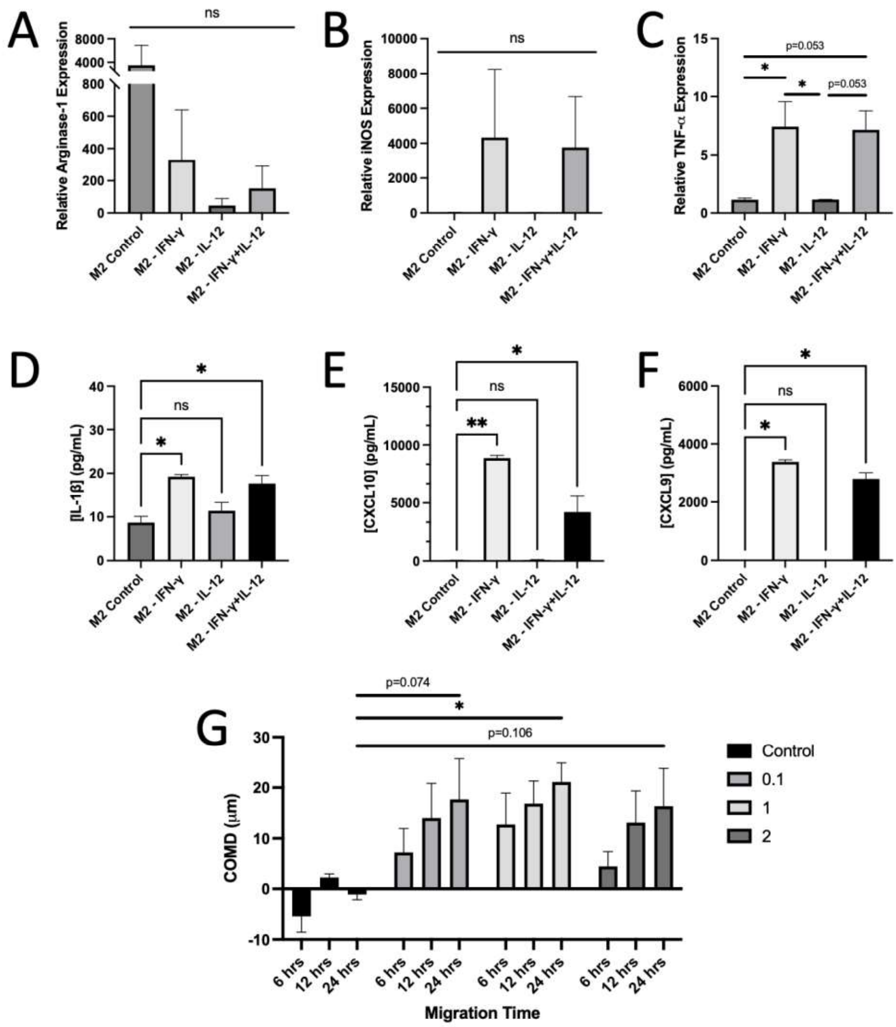

Supplemental Figure S9: Bone marrow derived macrophages were polarized to an M2-like phenotype via IL-4 and IL-13 cytokines and then treated with either IFN-γ, IL-12, or a combination of the two. RT-qPCR revealed an increase in inflammatory markers (**B,C**) and decrease in M2 marker Arg-1 (**A**) for those treated with either IFN-γ alone or the combination. (**D-F**) Luminex panels showed an increase in inflammatory cytokines for those treated with either IFN-γ alone or the combination. (**G**) Chemotaxis assay revealed the effectiveness of CCL2 at causing migration in M2-like macrophages, as measured by the center of mass distance (COMD). N=3, data presented as mean +/- SD, $^*p<0.05$.

Supplemental Figure S10: BMDM Cell Viability after Cryogel Treatment

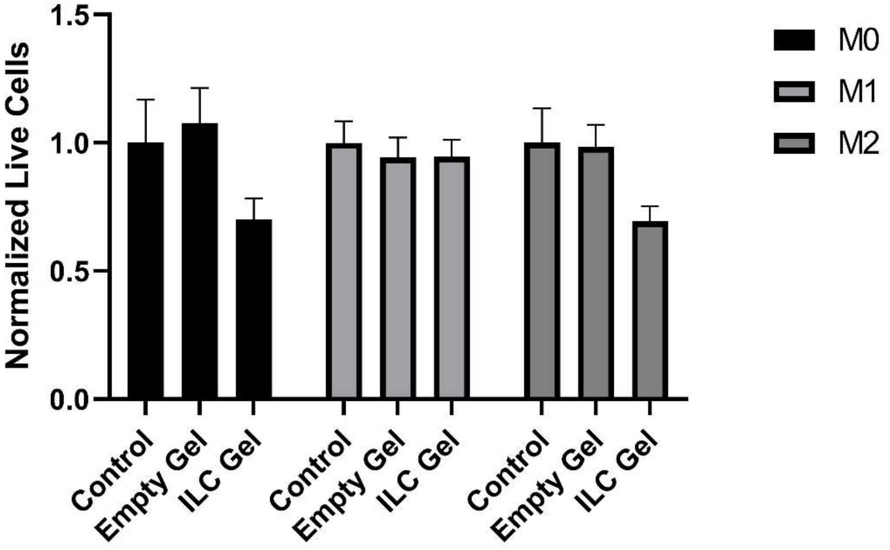

Supplemental Figure S10: BMDMs treated with cryogels for 48 hours did not exhibit any significant decreases in viability. N=4, data presented as mean +/- SD

Supplemental Figure S11: Evaluation of BMDM repolarization after treatment with ILC Gels

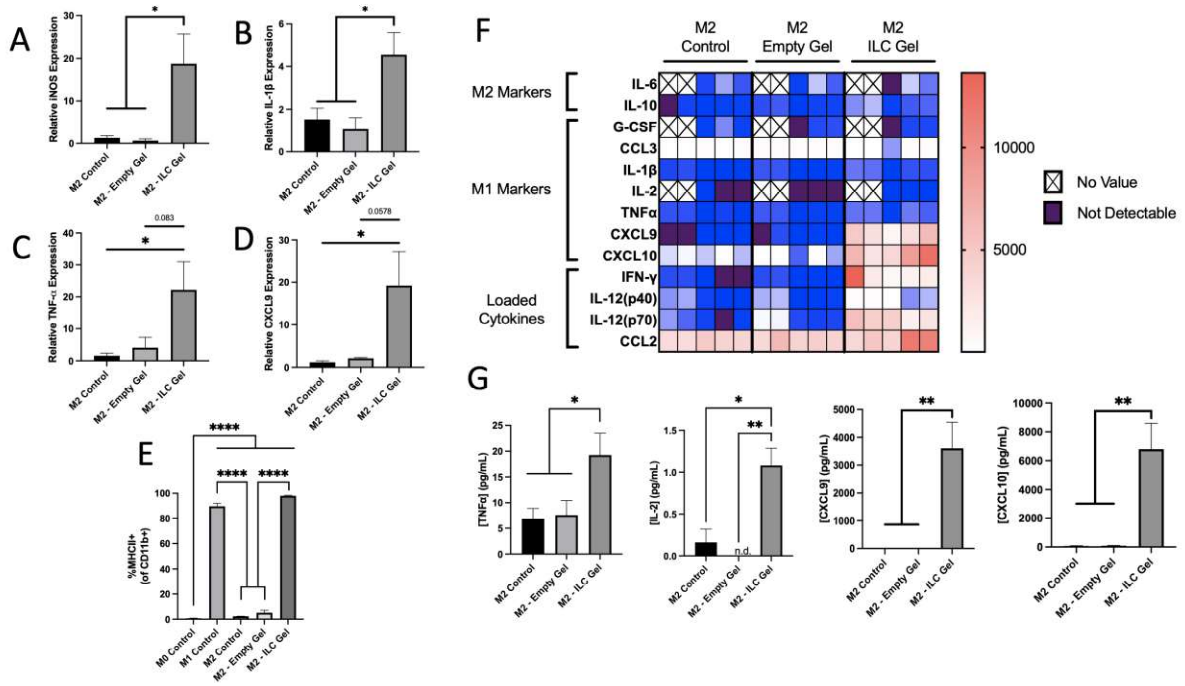

Supplemental Figure S11: M2-polarized BMDMs were treated with Empty Gels or ILC Gels for 48 hours. qRT-PCR analysis of RNA expression revealed significant increases in (**A**) iNOS, (**B**) IL-1β, (**C**) TNF-α, and (**D**) CXCL9 in ILC-treated cells compared to both control and Empty Gel treatment. **(E)** Flow cytometry analysis revealed a significant increase in MHCII. (**F**) Luminex analysis of released cytokines and chemokines revealed large shifts to a functional M1 phenotype, including (**G**) significant increases in production of TNF-α, IL-2, CXCL9, and CXCL10. N=3, data presented as mean +/- SD, *p<0.05, **p<0.01, ***p<0.001, ****p<0.0001

Supplemental Figure S12: PyMT Explant *In Vitro* Viability

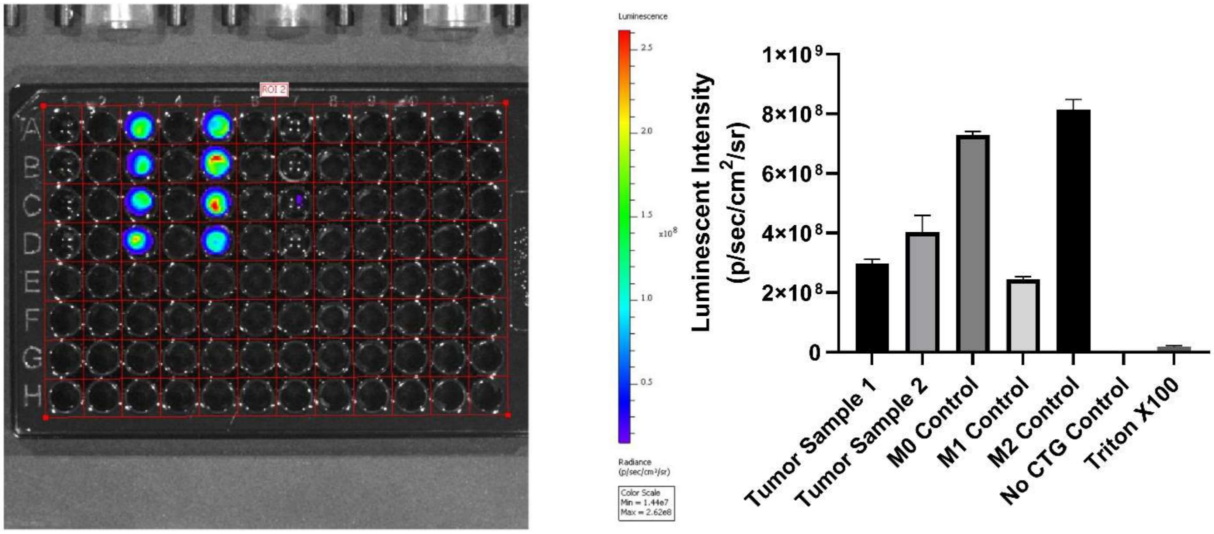

Supplemental Figure S12: PyMT tumor explants were cultured for 96 hours *in vitro* and then evaluated with a CellTiter-Glo viability assay to ensure live cells were present after 4 days of culture. N=4, data presented as mean +/- SD.

Supplemental Figure S13: Toxicity Panel and Mouse Weights

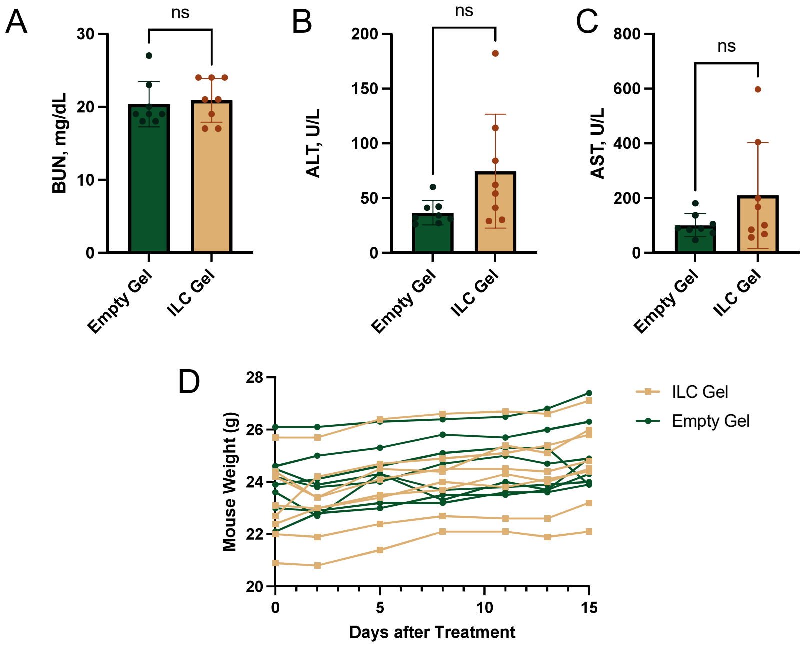

Supplemental Figure S13: Liver and kidney function of mice as a measure of BUN, ALT, and AST. Toxicity levels were not significantly different between empty gel group and ILC treatment group. Mouse weights after treatment did not vary between groups, indicating no obvious toxicity from the ILC treatment. N=8, data presented as mean +/- SD.
